# Supplementary figures and images for: Polygenic scores, diet quality, and type 2 diabetes risk: An observational study among 35,759 adults from 3 US cohorts
Source: PLoS Med. 2022 Apr 26;19(4):e1003972. doi: 10.1371/journal.pmed.1003972 (PMC9041832; doi:10.1371/journal.pmed.1003972)

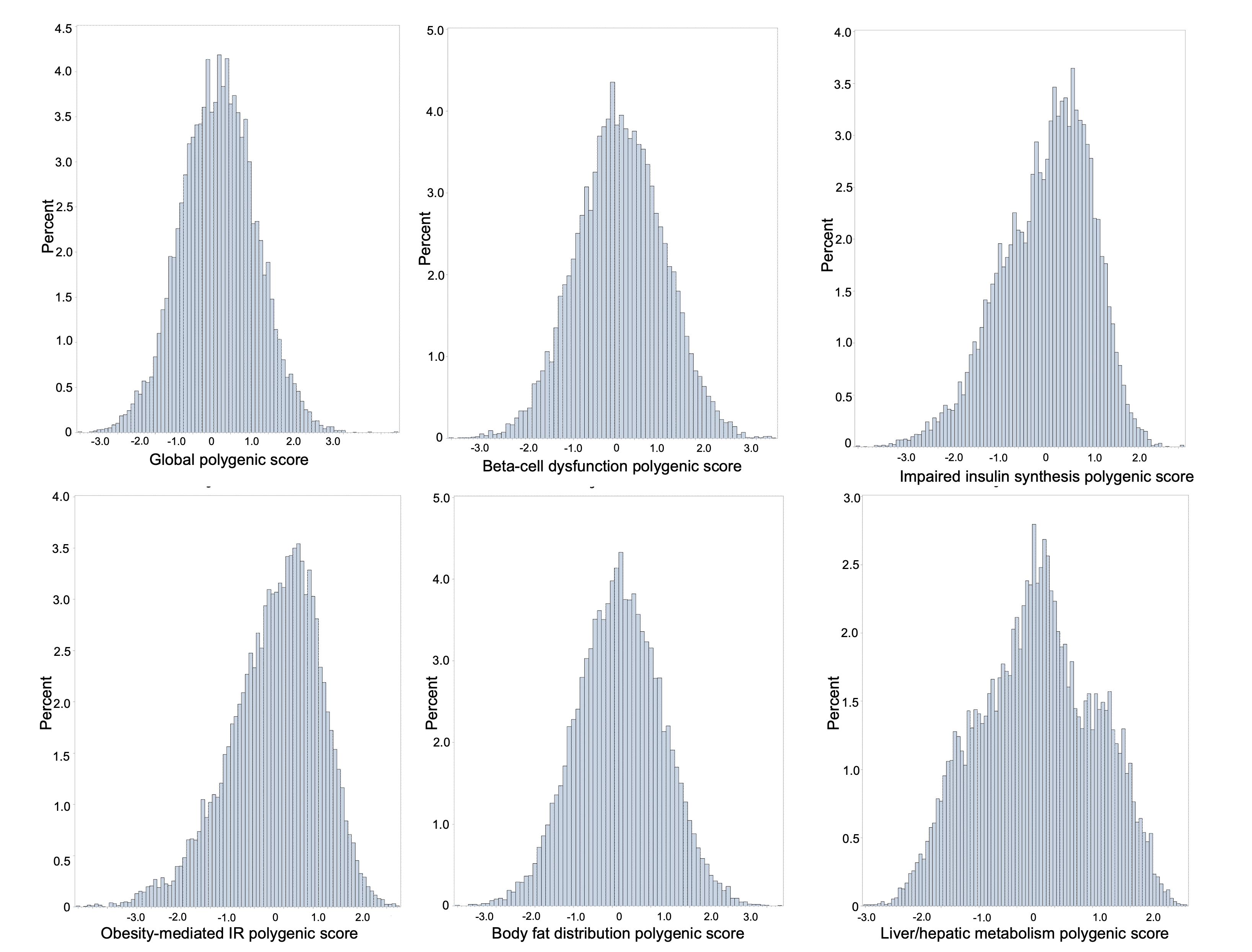

Supplement: S1 Fig — Distribution of the global and pathway polygenic scores in the NHS. NHS, Nurses’ Health Study. (PNG) [file pmed.1003972.s001.png]

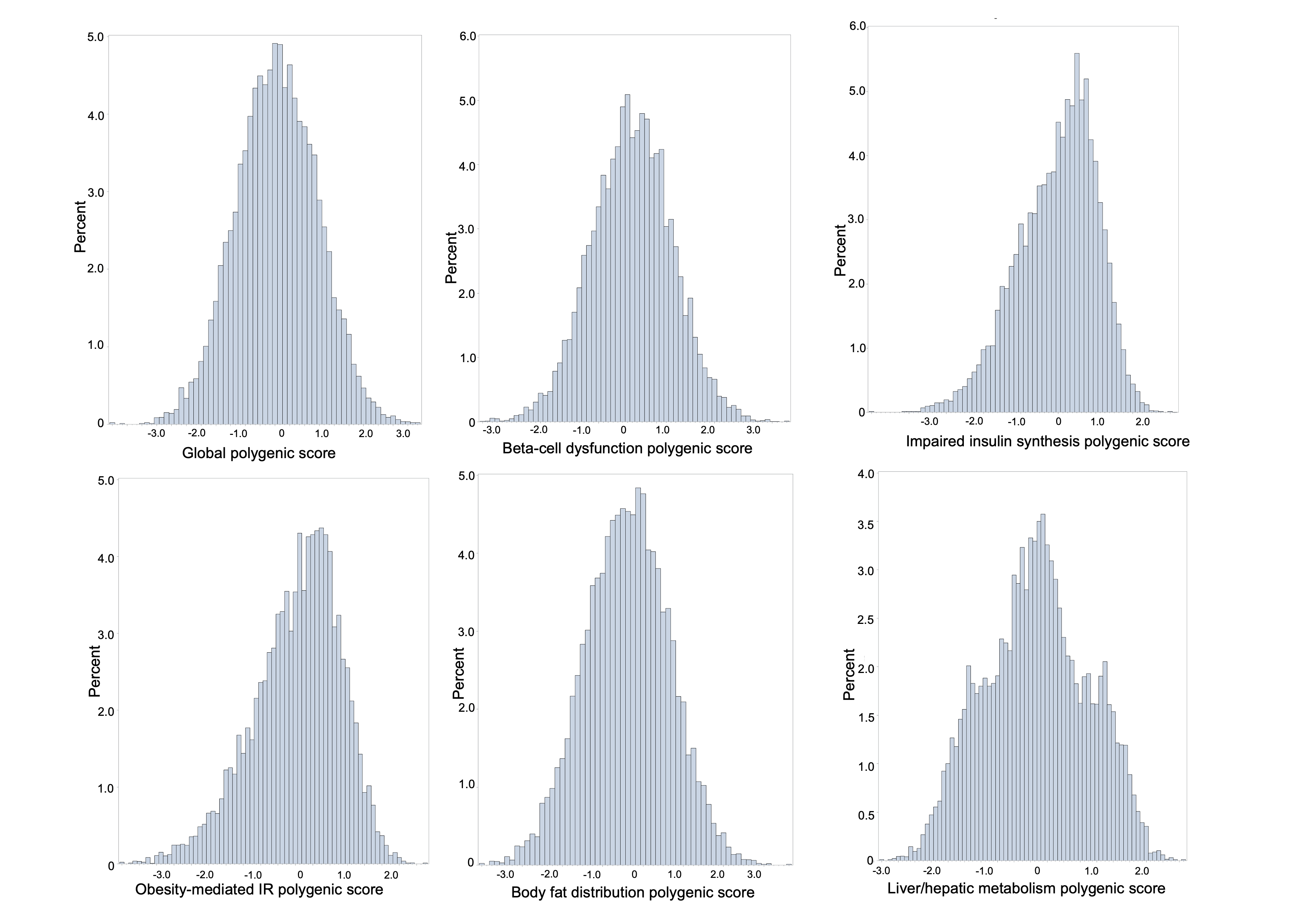

Supplement: S2 Fig — Distribution of the global and pathway polygenic scores in the HPFS. HPFS, Health Professionals Follow-up Study. (PNG) [file pmed.1003972.s002.png]

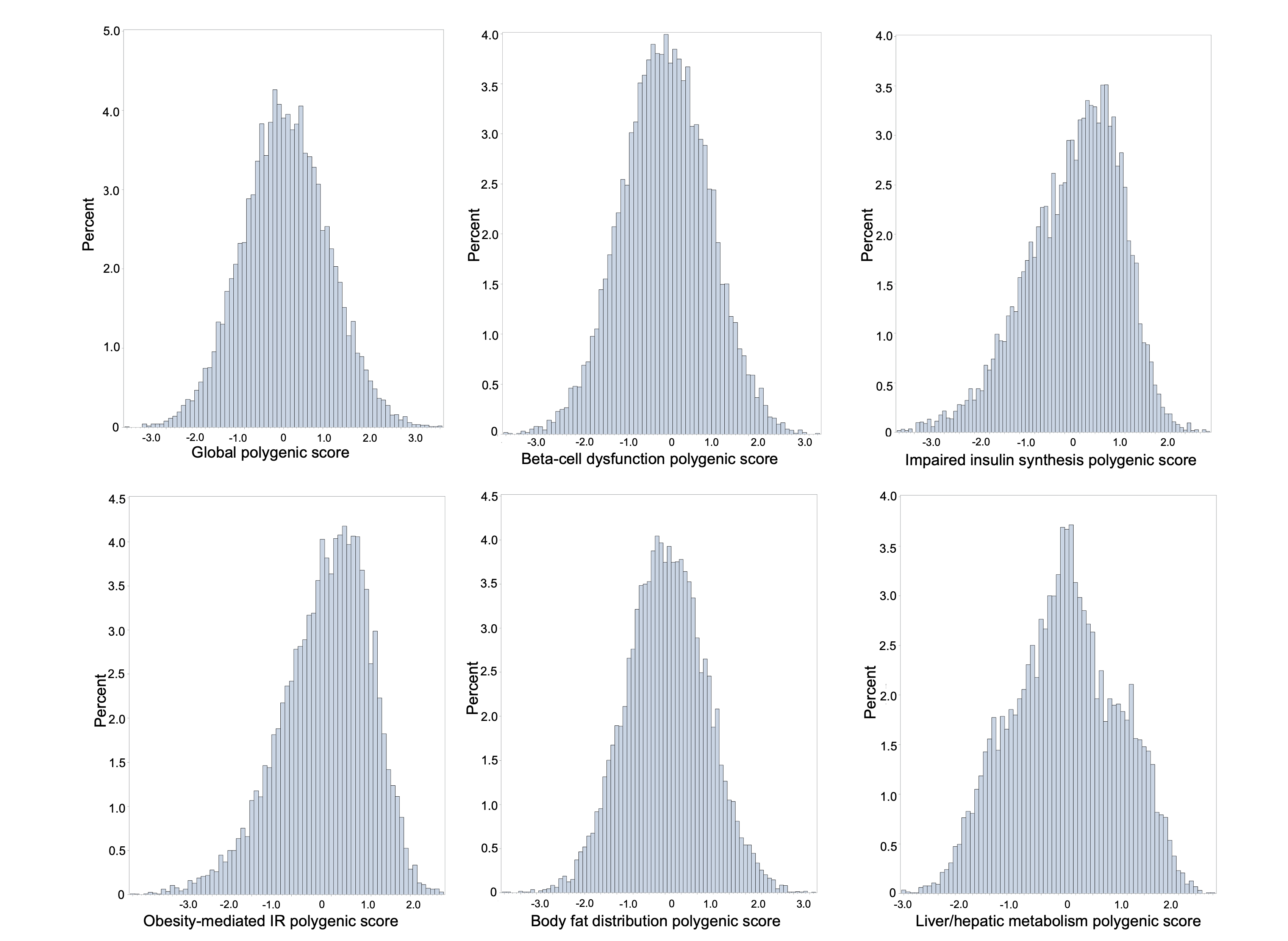

Supplement: S3 Fig — Distribution of the global and pathway polygenic scores in the NHS II. NHS, Nurses’ Health Study. (PNG) [file pmed.1003972.s003.png]

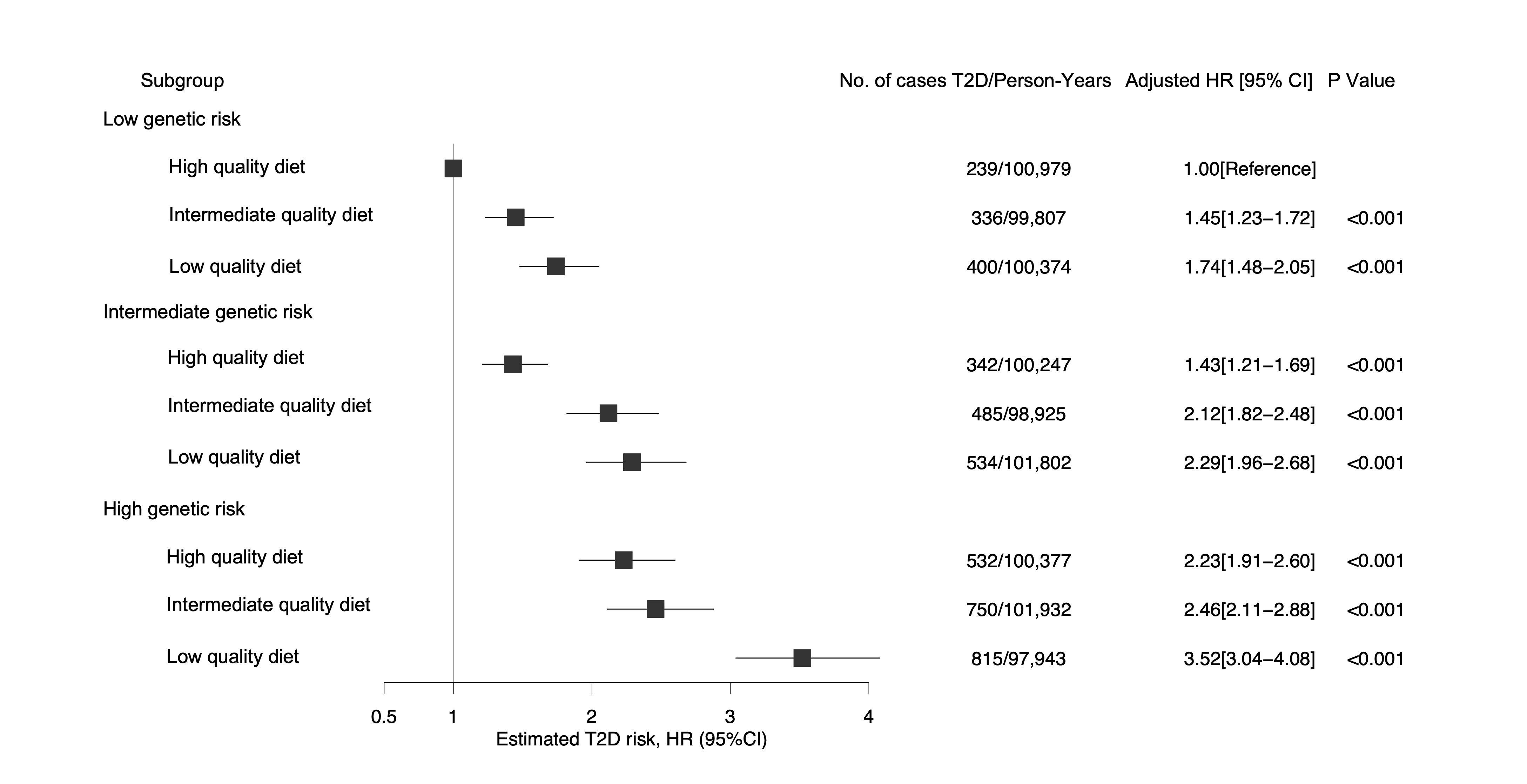

Supplement: S6 Fig — Shown are age-adjusted HRs and 95% CI of the estimate for type 2 diabetes according to genetic risk and diet quality categories using the AHEI score. In these comparisons, participants with low genetic risk and high-quality diet served as the reference group. Cox proportional hazards models were stratified by age and adjusted for ancestry-derived principal components (not time-varying). A fixed-effects meta-analysis was used to combine cohort-specific results. AHEI, Alternate Healthy Eating Index; CI, confidence interval; HR, hazard ratio. (PNG) [file pmed.1003972.s006.png]

**A**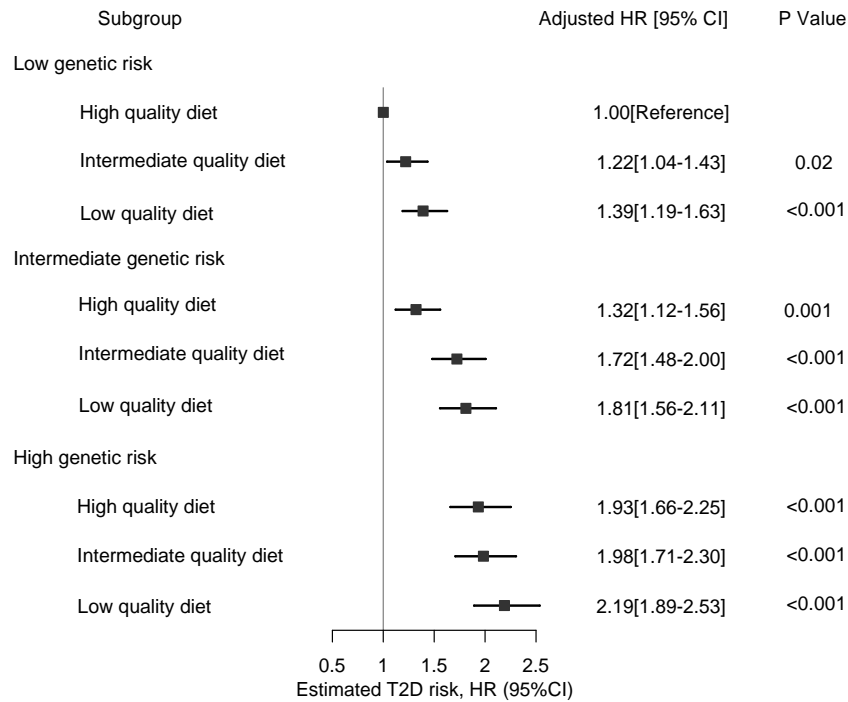**B**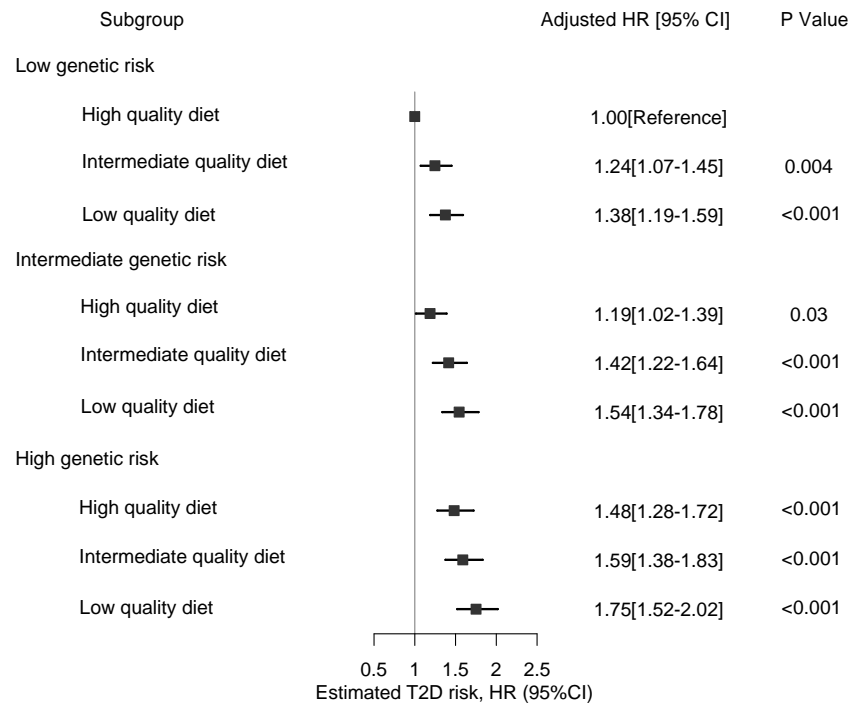

**C**

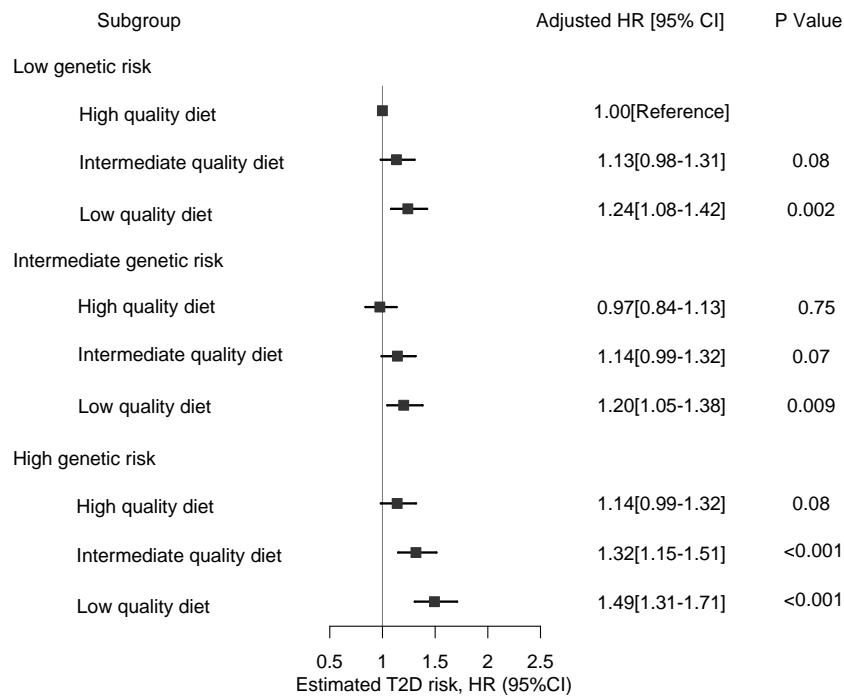

**D**

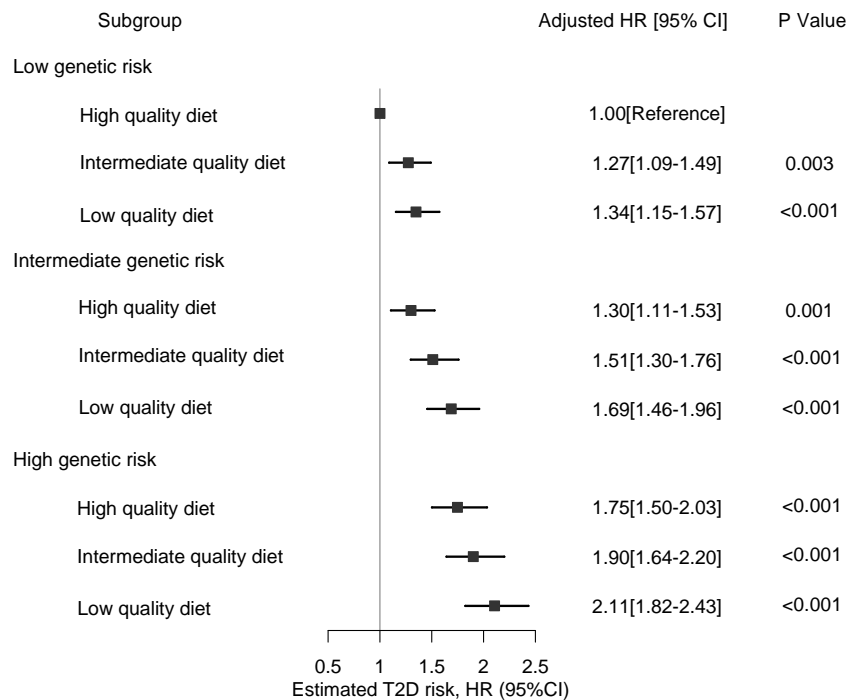

E

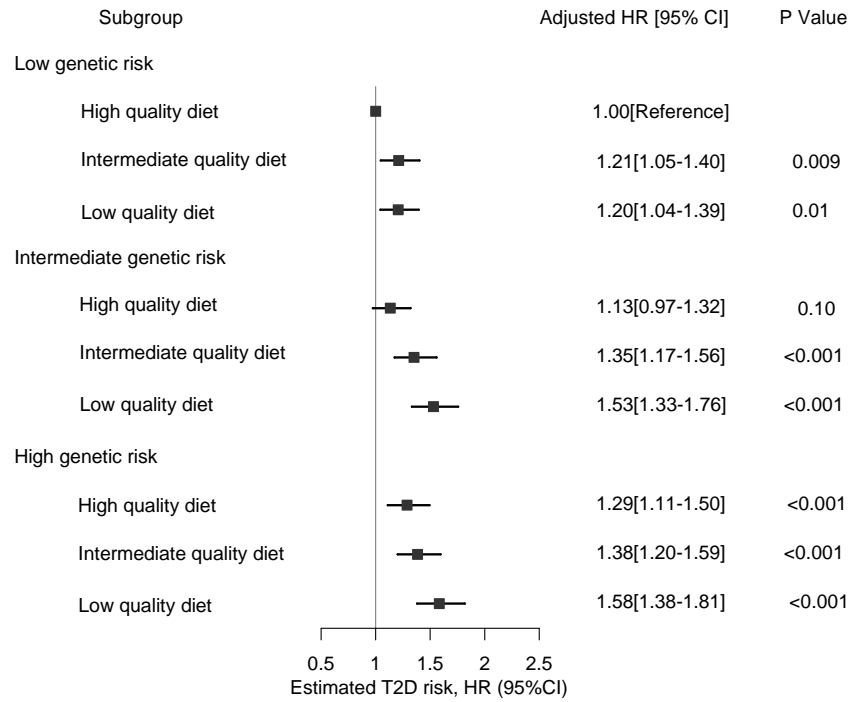

Supplement: S7 Fig — Shown are multivariable-adjusted HRs and 95% CI of the estimate for type 2 diabetes incidence according to pathway-specific polygenic score and diet quality categories. (A) Beta-cell polygenic score, (B) proinsulin polygenic score, (C) obesity polygenic score, (D) lipodystrophy polygenic score, and (E) liver metabolism polygenic score. In these comparisons, participants at low genetic risk with high-quality diet served as the reference group. A fixed-effects meta-analysis was used to combine cohort-specific results. CI, confidence interval; HR, hazard ratio. (PDF) [file pmed.1003972.s007.pdf]

**A**

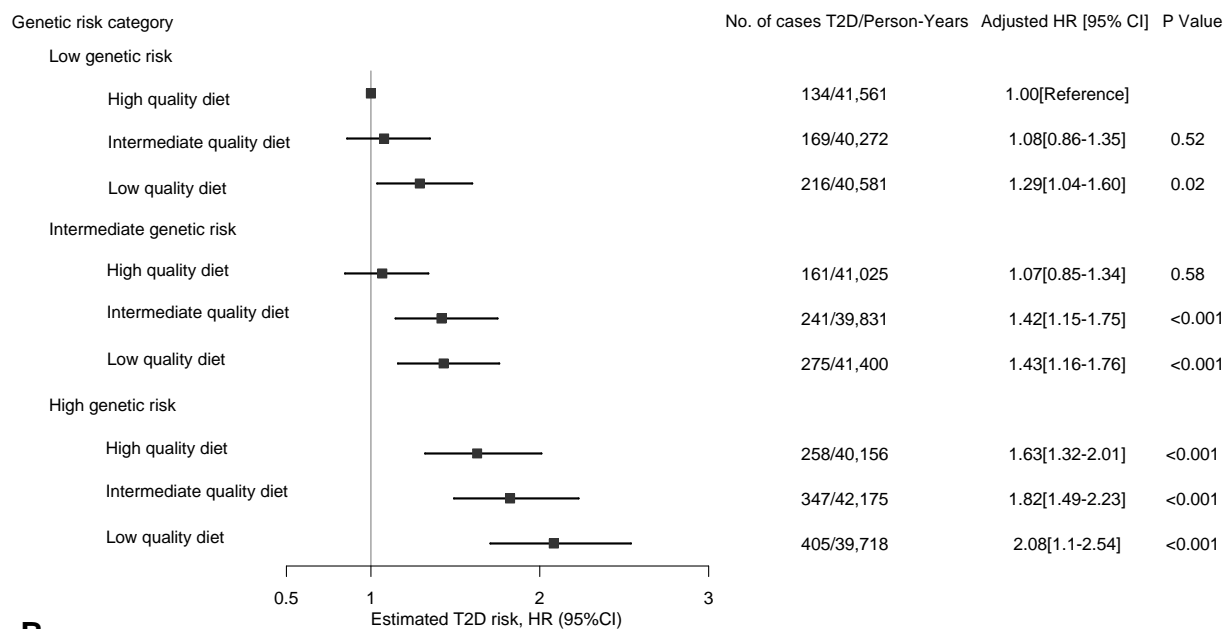

**B**

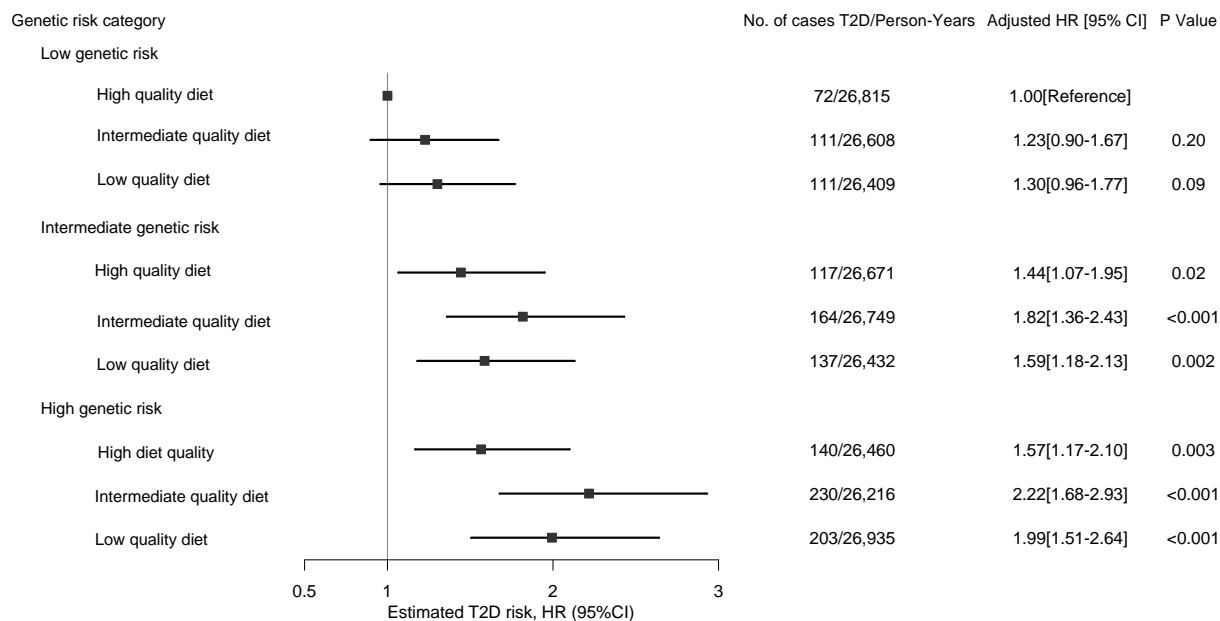

C

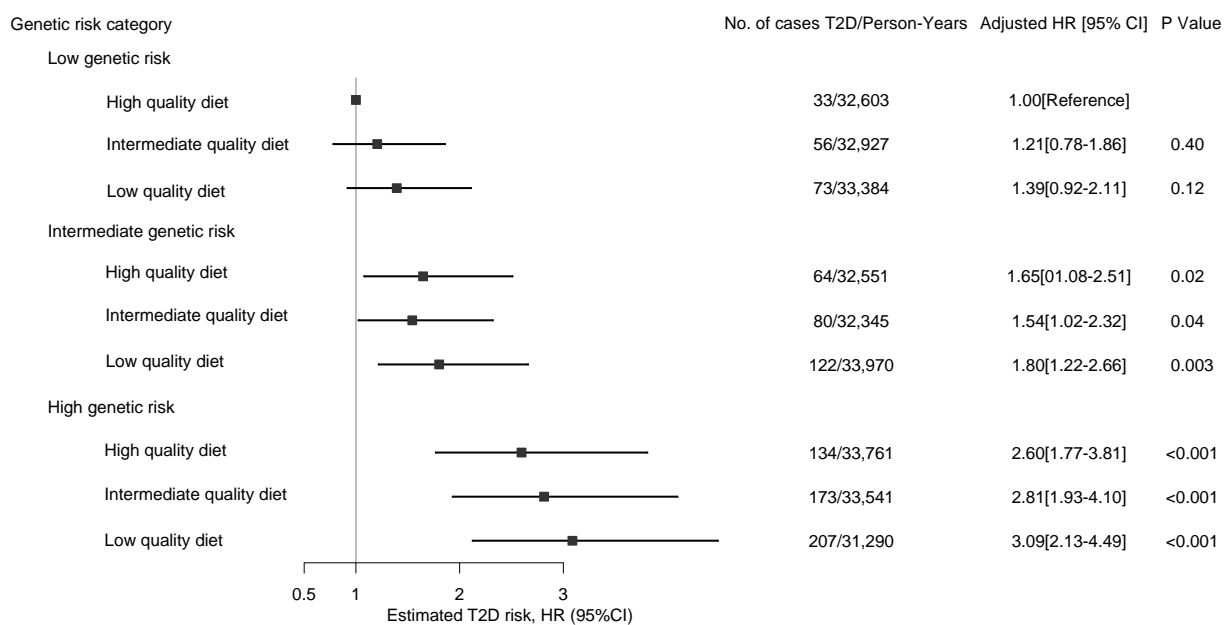

Supplement: S8 Fig — Shown are multivariable-adjusted HRs and 95% CI of the estimate for type 2 diabetes in (A) NHS, (B) HPFS, and (C) NHS II according to genetic risk and diet quality categories. In these comparisons, participants with low genetic risk and high-quality diet served as the reference group. CI, confidence interval; HR, hazard ratio; HPFS, Health Professionals Follow-up Study; NHS, Nurses’ Health Study. (PDF) [file pmed.1003972.s008.pdf]

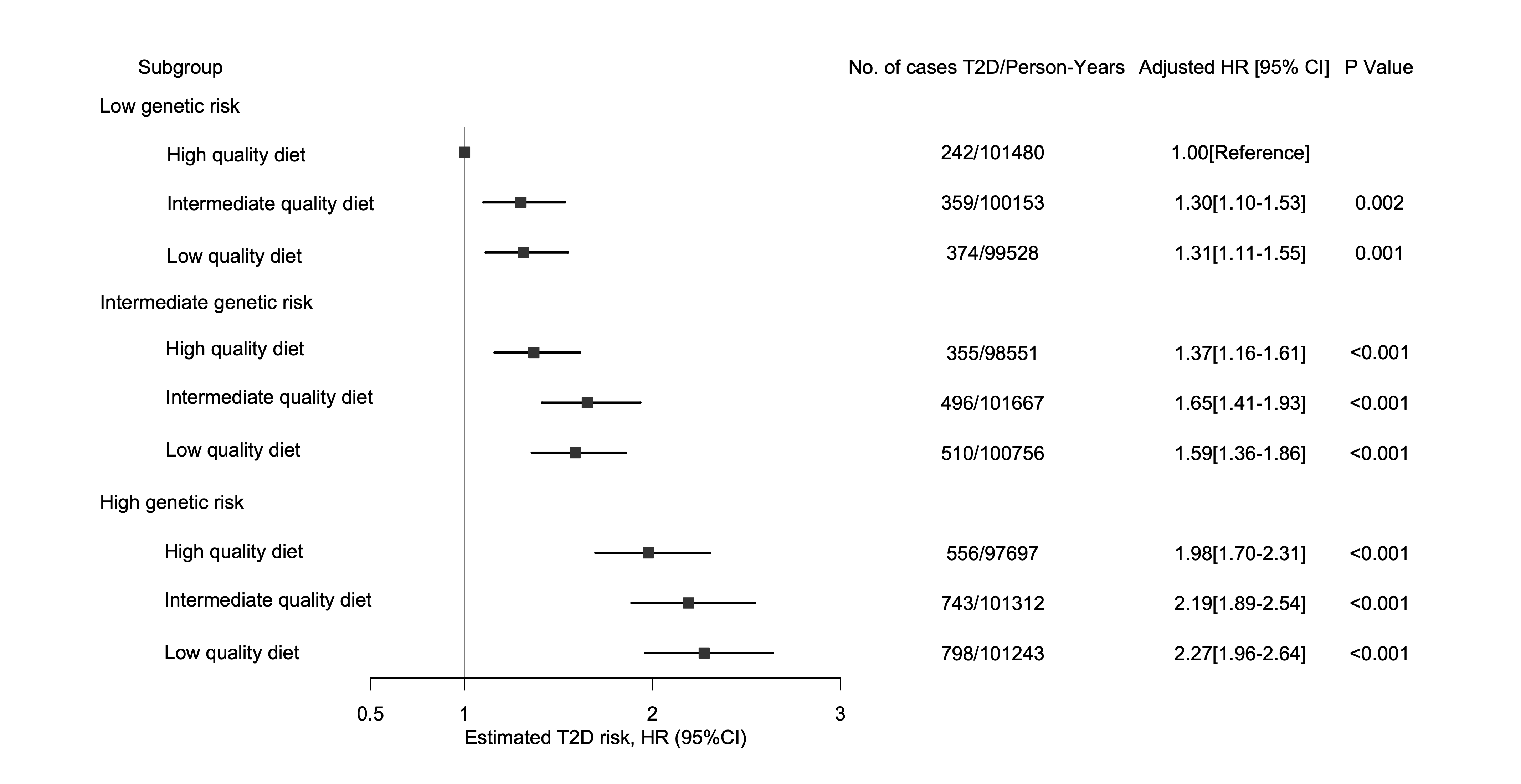

Supplement: S9 Fig — Shown are multivariable-adjusted HRs and 95% CI of the estimate for type 2 diabetes according to genetic risk and diet quality categories using the DASH score. In these comparisons, participants with low genetic risk and high-quality diet served as the reference group. A fixed-effects meta-analysis was used to combine cohort-specific results. CI, confidence interval; DASH, Dietary Approaches to Stop Hypertension; HR, hazard ratio. (PNG) [file pmed.1003972.s009.png]

**A**

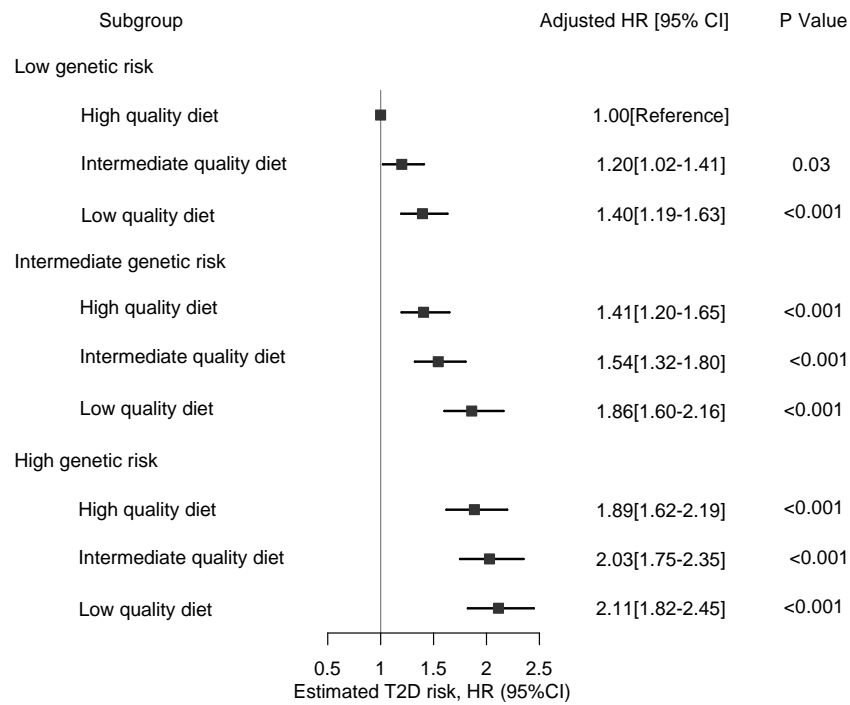

**B**

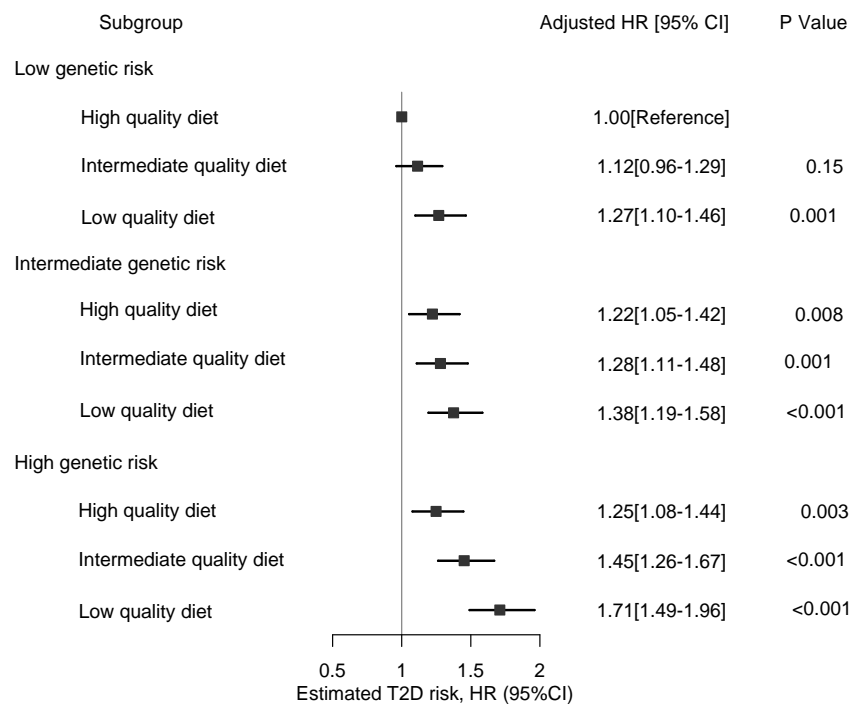

**C**

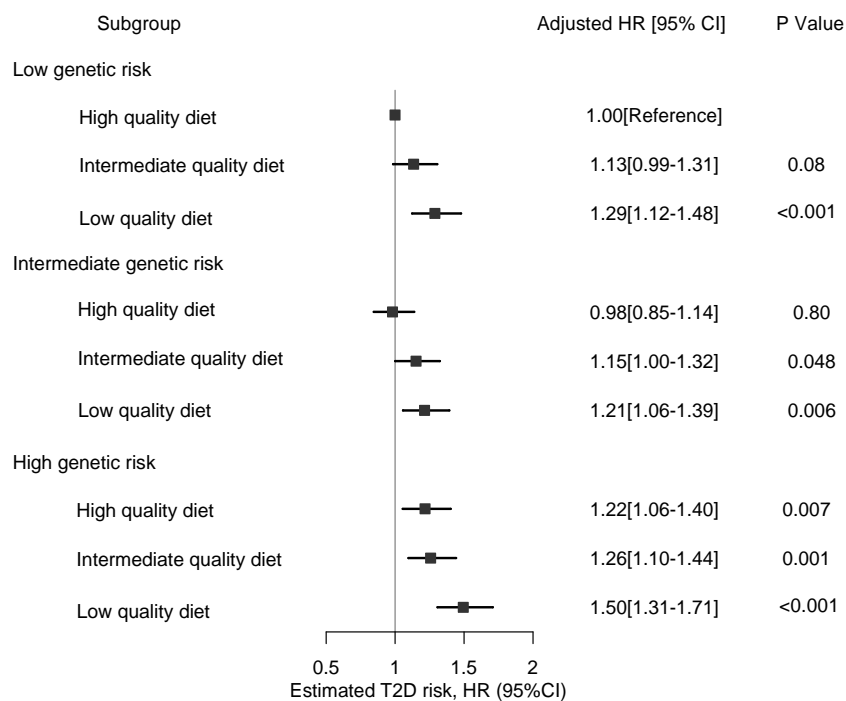

**D**

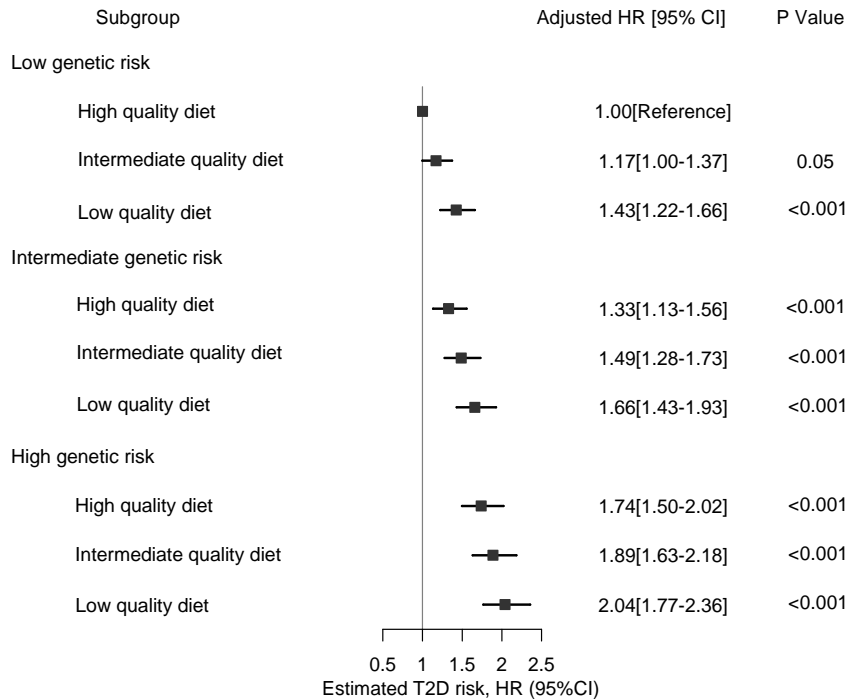

**E**

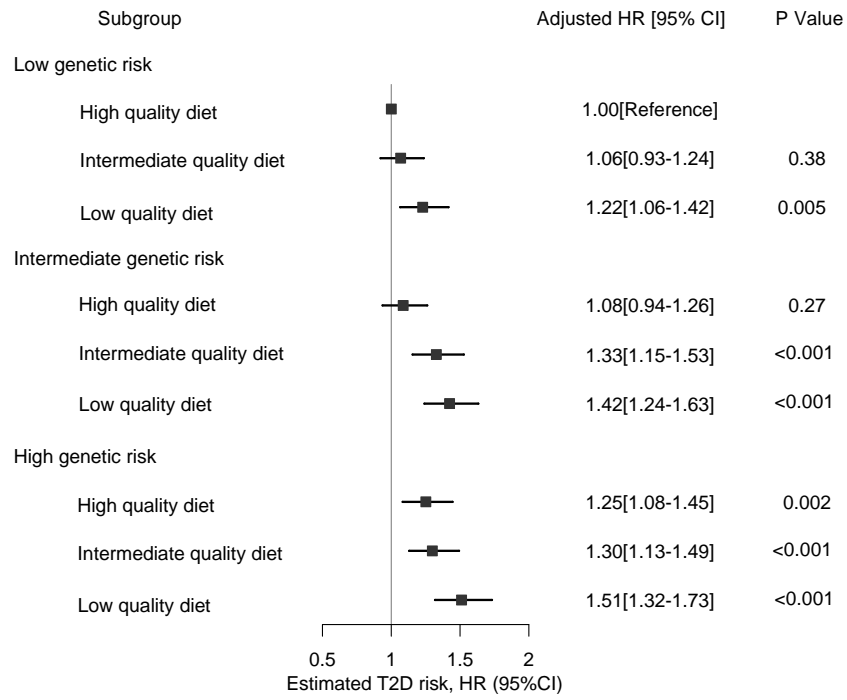

Supplement: S10 Fig — Shown are multivariable-adjusted HRs and 95% CI of the estimate for type 2 diabetes incidence according to pathway-specific polygenic score and diet quality categories using the DASH score. (A) Beta-cell polygenic score, (B) proinsulin polygenic score, (C) obesity polygenic score, (D) lipodystrophy polygenic score, and (E) liver metabolism polygenic score. In these comparisons, participants at low genetic risk with high-quality diet served as the reference group. A fixed-effects meta-analysis was used to combine cohort-specific results. CI, confidence interval; DASH, Dietary Approaches to Stop Hypertension; HR, hazard ratio. (PDF) [file pmed.1003972.s010.pdf]

**A**

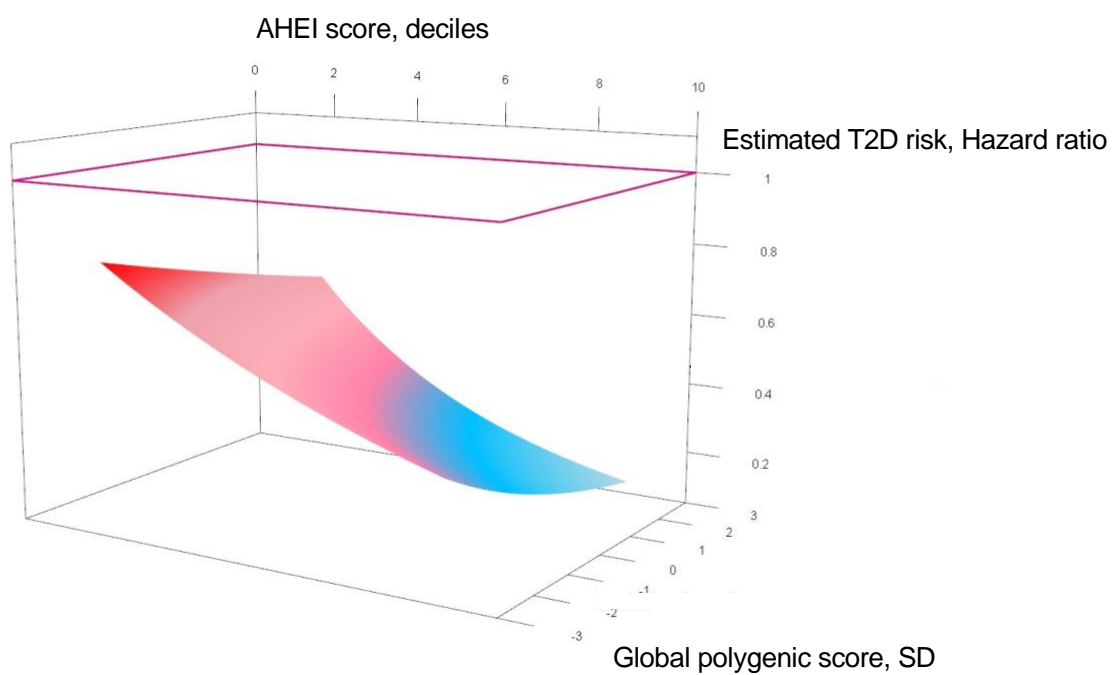

**B**

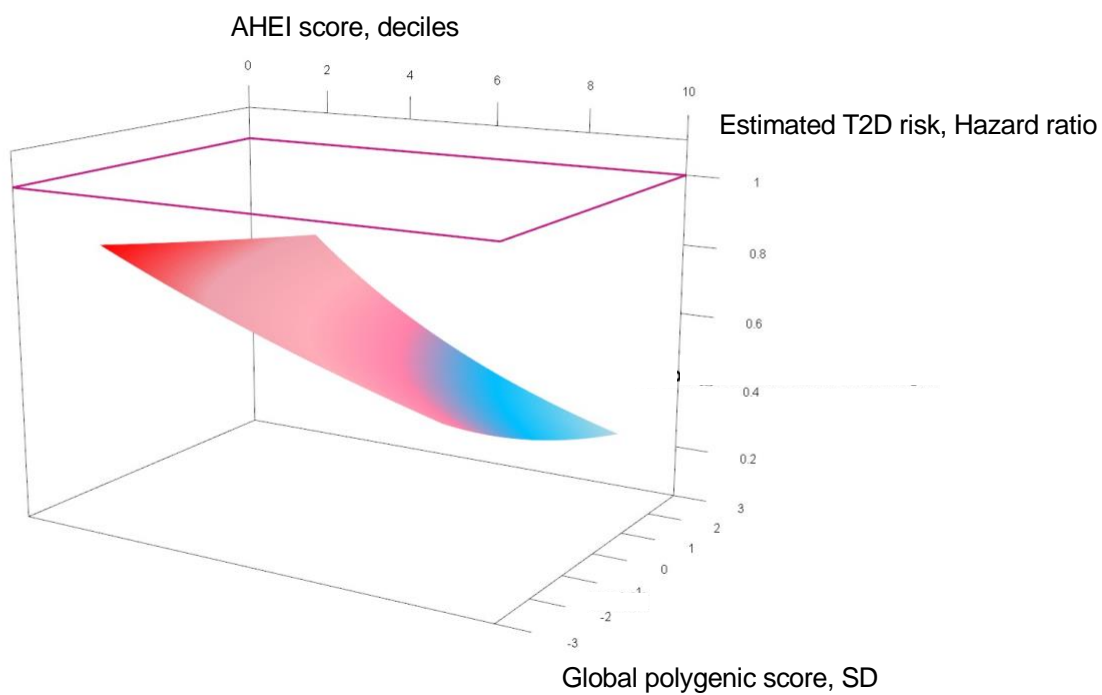

**C**

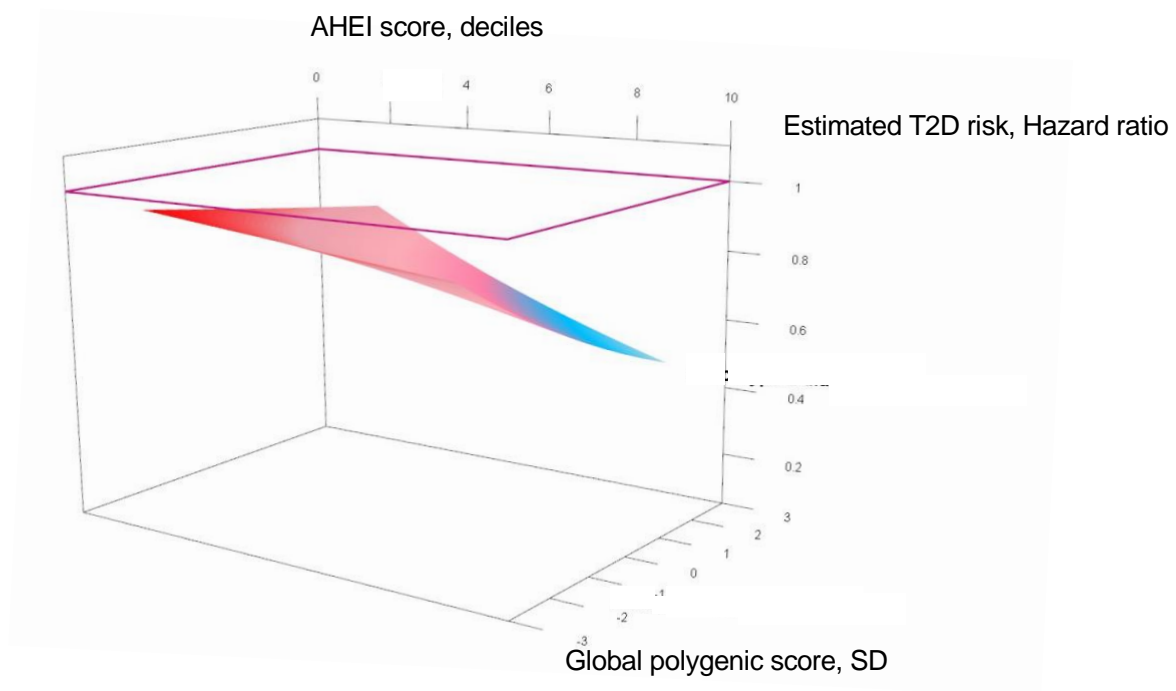

Supplement: S11 Fig — Three-dimensional illustrations of type 2 diabetes risk, genetic susceptibility, and diet quality by BMI among individuals with normal weight (A), overweight (B), and obese (C). The blue-colored region maps the lower risk area, and the red-colored area stands for higher risk area. Deciles of AHEI are inverse transformed, with 0 being good diet quality and 10 bad diet quality. Data from 3 cohorts were combined. Multivariate analyses were stratified by age and adjusted for time-varying confounders including cohort (not time-varying), ancestry-derived principal components (not time-varying), family history of diabetes (not time-varying), history of hypertension, history of hypercholesterolemia, menopausal status (women only), smoking status, physical activity, and total energy intake. P = 0.681 for 3-way interaction. AHEI, Alternate Healthy Eating Index; BMI, body mass index; SD, standard deviation; T2D, type 2 diabetes. (PDF) [file pmed.1003972.s011.pdf]
