# Supplementary material for: Polygenic scores, diet quality, and type 2 diabetes risk: An observational study among 35,759 adults from 3 US cohorts
Source: PLoS Med. 2022 Apr 26;19(4):e1003972. doi: 10.1371/journal.pmed.1003972 (PMC9041832; doi:10.1371/journal.pmed.1003972)
Supplement: S1 Table — (DOCX) [file pmed.1003972.s012.docx]

**S1 Table: Differences in baseline characteristics between the sample of participants included in this study and all participants in each original cohort.**

|  | **NHS participants** | | **HPFS participants** | | **NHS II participants** | |
| --- | --- | --- | --- | --- | --- | --- |
|  | **Included**  **(n=14,454)** | **Non-included**  **(n=121,701)** | **Included**  **(n=9,417)** | **Non-included**  **(n=51,529)** | **Included (n=11,888)** | **Non-included**  **(n=116,375)** |
| Age, years | 53 (7) | 53 (7) | 54 (9) | 54 (10) | 37 (4) | 36 (5) |
| Body mass index, mean (SD), kg/m^2^ | 25.2 (4.6) | 25.4 (4.8) | 25.5 (3.1) | 24.9 (5.1) | 24.3 (5) | 24.6 (5.4) |
| Smoking status |  |  |  |  |  |  |
| Current smoker, No. (%) | 2,479 (17.2) | 14,322 (18.2) | 728 (7.7) | 4,120 (7.9) | 1,245 (10.5) | 14,992 (11.8) |
| Disease prevalence |  |  |  |  |  |  |
| Hypertension, No. (%) | 2,193 (15.2) | 16,130 (13.3) | 1,833 (19.5) | 11,623 (22.6) | 345 (2.9) | 3,959 (3.4) |
| Dyslipidemia, No. (%) | 1,133 (7.8) | 8,242 (6.8) | 1,117 (11.9) | 6,585 (12.8) | 1,173 (9.9) | 10,446 (9.0) |
| Family history of diabetes, No. (%) | 4,323 (29.9) | 31,317 (25.7) | 2,695 (28.6) | 9,173 (22.6) | 4,294 (36.1) | 39,309 (33.8) |
| Total energy intake, mean (SD), kcal/day | 1,781 (520) | 1,766 (527) | 1,988 (557) | 1,986 (620) | 1,802 (535) | 1,789 (548) |
| AHEI score, mean (SD)^*^ | 52.1 (11.3) | 51.7 (11.2) | 52.6 (11.7) | 52.8 (11.5) | 48.9 (11) | 48.6 (11) |

**Table Legend:** Values are means (SD) for continuous variables; numbers and (percentages) for categorical variables. All values are standardized to the age distribution of the study population. The study baseline was set at 1986 for the Nurses’ Health Study I (NHS) and the Health Professionals Follow-up Study (HPFS) and 1991 for the Nurses’ Health Study II (NHS II).

MET denotes metabolic equivalent tasks.

^*^ Scores on the Alternate Healthy Eating Index (AHEI) range from 0 to 110, with higher scores indicating a healthy diet.
