# Supplementary material for: Polygenic scores, diet quality, and type 2 diabetes risk: An observational study among 35,759 adults from 3 US cohorts
Source: PLoS Med. 2022 Apr 26;19(4):e1003972. doi: 10.1371/journal.pmed.1003972 (PMC9041832; doi:10.1371/journal.pmed.1003972)
Supplement: S2 Table — (DOCX) [file pmed.1003972.s013.docx]

**S2 Table: Characteristics of genetic variants used to build the five different pathway-specific polygenic scores.**

| **Beta cell dysfunction polygenic score** | | | | | |
| --- | --- | --- | --- | --- | --- |
| **Loci** | **SNP** | **ID** | **EA** | **EAF** | **Weight** |
| *MTNR1B* | rs10830961 | 11_92694757_A_G | G | 0.38 | 3.81 |
| *CDKAL1* | rs7756992 | 6_20679709_A_G | G | 0.25 | 3.05 |
| *C2CD4A* | rs4502156 | 15_62383155_T_C | T | 0.52 | 2.68 |
| *HHEX* | rs1111875 | 10_94462882_C_T | C | 0.56 | 2.66 |
| *TCF7L2* | rs7903146 | 10_114758349_C_T | T | 0.25 | 2.47 |
| *SLC30A8* | rs3802177 | 8_118185025_G_A | G | 0.75 | 2.41 |
| *CDKN2A_B* | rs10811661 | 9_22134094_T_C | T | 0.79 | 1.77 |
| *CDC123.CAMK1D* | rs11257655 | 10_12307894_C_T | T | 0.28 | 1.59 |
| *HNF1A* | rs7957197 | 12_121460686_T_A | T | 0.85 | 1.56 |
| *AP3S2* | rs1371135 | 15_90391270_T_C | T | 0.32 | 1.54 |
| *ZHX3* | rs17265513 | 20_39832628_T_C | C | 0.20 | 1.37 |
| *UBE2E2* | rs1496653 | 3_23454790_A_G | A | 0.81 | 1.35 |
| *ACSL1* | rs1996546 | 4_185714289_G_T | G | 0.85 | 1.35 |
| *PRC1* | rs12899811 | 15_91544076_A_G | G | 0.23 | 1.26 |
| *GIPR* | rs8108269 | 19_46158513_T_G | G | 0.28 | 1.25 |
| *GLP2R* | rs17676067 | 17_9791375_T_C | C | 0.47 | 1.22 |
| *KCNJ11* | rs5215 | 11_17408630_C_T | C | 0.41 | 1.19 |
| *KCNQ1_2* | rs233449 | 11_2843803_G_A | G | 0.73 | 1.18 |
| *ABO* | rs505922 | 9_136149229_T_C | C | 0.32 | 1.17 |
| *ANK1* | rs516946 | 8_41519248_T_C | C | 0.82 | 1.12 |
| *GLIS3* | rs10758593 | 9_4292083_G_A | A | 0.43 | 1.09 |
| *HNF1B* | rs4430796 | 17_36098040_G_A | G | 0.53 | 1.04 |
| *CTRB2* | rs9921586 | 16_75245003_G_T | G | 0.86 | 1.01 |
| *CDKN2A_2* | rs2184061 | 9_22061562_C_A | A | 0.61 | 0.97 |
| *DUSP8* | rs2334499 | 11_1696849_C_T | T | 0.44 | 0.96 |
| *ADCY5* | rs11708067 | 3_123065778_A_G | A | 0.78 | 0.92 |
| *GIP* | rs15563 | 17_47005193_A_G | G | 0.53 | 0.88 |
| *HNF4A* | rs4812829 | 20_42989267_G_A | A | 0.18 | 0.84 |
| *HSD17B12* | rs3736505 | 11_43876435_A_G | G | 0.30 | 0.78 |
| *TLE4* | rs17791513 | 9_81905590_A_G | A | 0.93 | 0.78 |
| **Impaired insulin synthesis polygenic score** | | | | | |
| **Loci** | **SNP** | **ID** | **EA** | **EAF** | **Weight** |
| *ARAP1* | rs1552224 | 11_72433098_A_C | A | 0.88 | 4.48 |
| *SPRY2* | rs1359790 | 13_80717156_G_A | G | 0.73 | 1.07 |
| *DGKB_2* | rs10276674 | 7_14922007_T_C | C | 0.22 | 1.00 |
| *IGF2BP2* | rs4402960 | 3_185511687_G_T | T | 0.29 | 0.97 |
| *CCND2* | rs11063069 | 12_4374373_A_G | G | 0.21 | 0.81 |
| *HNF4A* | rs4812829 | 20_42989267_G_A | A | 0.18 | 0.79 |
| *CDC123.CAMK1D* | rs11257655 | 10_12307894_C_T | T | 0.28 | 0.78 |
| *ARAP1* | rs1552224 | 11_72433098_A_C | A | 0.88 | 4.48 |
| **Obesity polygenic score** | | | | | |
| **Loci** | **SNP** | **ID** | **EA** | **EAF** | **Weight** |
| *FTO* | rs9939609 | 16_53820527_T_A | A | 0.45 | 4.96 |
| *MC4R* | rs12970134 | 18_57884750_G_A | A | 0.31 | 2.78 |
| *NRXN3* | rs10146997 | 14_79945162_A_G | G | 0.21 | 1.72 |
| *HSD17B12* | rs3736505 | 11_43876435_A_G | G | 0.30 | 1.24 |
| *RBMS1* | rs6742799 | 2_161317460_A_C | A | 0.83 | 1.04 |
| **Body fat distribution polygenic score** | | | | | |
| **Loci** | **SNP** | **ID** | **EA** | **EAF** | **Weight** |
| *IRS1* | rs2943641 | 2_227093745_T_C | C | 0.61 | 3.01 |
| *GRB14* | rs13389219 | 2_165528876_C_T | C | 0.56 | 2.74 |
| *PPARG* | rs1801282 | 3_12393125_C_G,A | C | 0.93 | 2.11 |
| *LYPLAL1* | rs2820443 | 1_219753509_T_C | T | 0.68 | 1.92 |
| *ANKRD55* | rs459193 | 5_55806751_A_G | G | 0.78 | 1.80 |
| *CMIP* | rs2925979 | 16_81534790_T_C | T | 0.29 | 1.74 |
| *KLF14* | rs3996352 | 7_130444934_A_G | A | 0.55 | 1.63 |
| *LPL* | rs10503669 | 8_19847690_C_A | C | 0.88 | 1.54 |
| *ANKRD55_2* | rs3843467 | 5_55856375_G_T | T | 0.19 | 1.31 |
| *ARL15* | rs702634 | 5_53271420_G_A | A | 0.73 | 1.29 |
| *ADCY5* | rs11708067 | 3_123065778_A_G | A | 0.78 | 1.24 |
| *C17orf58* | rs9891146 | 17_65988049_T_C | T | 0.26 | 1.08 |
| *POU5F1* | rs3132524 | 6_31136714_T_C | C | 0.70 | 1.06 |
| *MACF1* | rs2296172 | 1_39835817_A_G | G | 0.18 | 1.03 |
| *ZBED3* | rs4457053 | 5_76424949_G_A | G | 0.28 | 0.96 |
| *KIF9* | rs2276853 | 3_47282303_G_A | A | 0.61 | 0.96 |
| *ADAMTS9* | rs6795735 | 3_64705365_C_T | C | 0.54 | 0.95 |
| *CCND2* | rs11063069 | 12_4374373_A_G | G | 0.21 | 0.94 |
| *FAF1* | rs17106184 | 1_50909985_G_A | G | 0.89 | 0.81 |
| *MPHOSPH9* | rs1106240 | 12_123626982_C_T | T | 0.80 | 0.79 |
| **Lipid/hepatic metabolism polygenic score** | | | | | |
| **Loci** | **SNP** | **ID** | **EA** | **EAF** | **Weight** |
| *GCKR* | rs780094 | 2_27741237_T_C | C | 0.62 | 5.34 |
| *CILP2* | rs16996148 | 19_19658472_G_T | T | 0.08 | 2.05 |
| *HLA.DQA1* | rs9271775 | 6_32594328_C_T | T | 0.76 | 0.98 |
| *PNPLA3* | rs738409 | 22_44324727_C_G | *G* | 0.23 | 0.84 |
| *TSPAN8.LGR5* | rs7961581 | 12_71663102_C_T | C | 0.23 | 0.83 |

**Table Legend:** List of type 2 diabetes variants used to build each pathway-specific polygenic score. Genetic variants and weights obtained from elsewhere [1]. The number of genetic variants included in each score in the original publication were the same as included in this study as proxies were used for missing genotypes. Abbreviations: SNP, Single nucleotide polymorphism; ID, Identification based on hg19.; EA, effect allele associated with type 2 diabetes; EAF, effect allele frequency.

**Reference**

1. Udler MS, Kim J, von Grotthuss M, Bonàs-Guarch S, Cole JB, Chiou J, et al. Type 2 diabetes genetic loci informed by multi-trait associations point to disease mechanisms and subtypes: A soft clustering analysis. PLoS Med. 2018;15(9):e1002654
