# Supplementary material for: Polygenic scores, diet quality, and type 2 diabetes risk: An observational study among 35,759 adults from 3 US cohorts
Source: PLoS Med. 2022 Apr 26;19(4):e1003972. doi: 10.1371/journal.pmed.1003972 (PMC9041832; doi:10.1371/journal.pmed.1003972)
Supplement: S3 Table — (DOCX) [file pmed.1003972.s014.docx]

**S3 Table: Associations of global and process-specific polygenic scores with type 2 diabetes risk in secondary analyses.**

|  | **Nurses’ Health Study** | | **Health Professionals Follow-up Study** | | **Nurses’ Health Study II** | | **Combined** | |
| --- | --- | --- | --- | --- | --- | --- | --- | --- |
|  | **HR (95%CI)** | ***P* Value** | **HR (95%CI)** | ***P* Value** | **HR (95%CI)** | ***P* Value** | **HR (95%CI)** | ***P* Value** |
| **Global polygenic score** |  |  |  |  |  |  |  |  |
| Crude model | 1.36 (1.30, 1.42) | <0.001 | 1.34 (1.27, 1.42) | <0.001 | 1.69 (1.58, 1.80) | <0.001 | 1.42 (1.38, 1.46) | <0.001 |
| Multivariable-adjusted w/ BMI | 1.28 (1.23, 1.35) | <0.001 | 1.27 (1.20, 1.35) | <0.001 | 1.51 (1.42, 1.62) | <0.001 | 1.33 (1.29, 1.37) | <0.001 |
| **Pathway-specific polygenic scores** |  |  |  |  |  |  |  |  |
| Beta-cell dysfunction |  |  |  |  |  |  |  |  |
| Crude model | 1.23 (1.18, 1.28) | <0.001 | 1.30 (1.23, 1.38) | <0.001 | 1.30 (1.22, 1.39) | <0.001 | 1.26 (1.23, 1.30) | <0.001 |
| Multivariable-adjusted w/ BMI | 1.21 (1.16, 1.27) | <0.001 | 1.30 (1.22, 1.37) | <0.001 | 1.28 (1.20, 1.37) | <0.001 | 1.25 (1.21, 1.29) | <0.001 |
| Impaired insulin synthesis |  |  |  |  |  |  |  |  |
| Crude model | 1.13 (1.08, 1.18) | <0.001 | 1.13 (1.07, 1.20) | <0.001 | 1.17 (1.10, 1.26) | <0.001 | 1.14 (1.11, 1.18) | <0.001 |
| Multivariable-adjusted w/ BMI | 1.12 (1.08, 1.17) | <0.001 | 1.14 (1.07, 1.21) | <0.001 | 1.16 (1.09, 1.24) | <0.001 | 1.13 (1.10, 1.17) | <0.001 |
| Obesity-mediated insulin resistance |  |  |  |  |  |  |  |  |
| Crude model | 1.07 (1.02, 1.11) | 0.003 | 1.13 (1.07, 1.20) | <0.001 | 1.09 (1.02, 1.17) | 0.011 | 1.09 (1.06, 1.13) | <0.001 |
| Multivariable-adjusted w/ BMI | 1.05 (1.01, 1.07) | 0.021 | 1.13 (1.07, 1.20) | <0.001 | 1.07 (1.00, 1.15) | 0.048 | 1.08 (1.04, 1.11) | <0.001 |
| Body fat distribution |  |  |  |  |  |  |  |  |
| Crude model | 1.24 (1.18, 1.29) | <0.001 | 1.24 (1.18, 1.32) | <0.001 | 1.28 (1.20, 1.36) | <0.001 | 1.25 (1.21, 1.29) | <0.001 |
| Multivariable-adjusted w/ BMI | 1.21 (1.16, 1.26) | <0.001 | 1.25 (1.18, 1.33) | <0.001 | 1.23 (1.15, 1.31) | <0.001 | 1.22 (1.19, 1.26) | <0.001 |
| Lipid/hepatic metabolism |  |  |  |  |  |  |  |  |
| Crude model | 1.15 (1.07, 1.20) | <0.001 | 1.11 (1.03, 1.18) | 0.004 | 1.11 (1.03, 1.20) | 0.008 | 1.13 (1.09, 1.17) | <0.001 |
| Multivariable-adjusted w/ BMI | 1.12 (1.08, 1.17) | <0.001 | 1.10 (1.04, 1.17) | 0.001 | 1.10 (1.03, 1.18) | 0.004 | 1.11 (1.08, 1.15) | <0.001 |

**Table Legend:** Hazards ratios and 95% confidence interval of the estimate for type 2 diabetes risk per SD increase in polygenic scores in each of the three prospective cohorts separately and in a combined analysis. Cox regression models were stratified by age (in months, continuous) and adjusted for ancestry-derived principal components (1 to 4) (crude model). The multivariable adjusted model without BMI was further adjusted for time-dependent confounders including family history of diabetes (not time-dependent, yes or no), hypertension (yes or no), hypercholesterolemia (yes or no), menopausal status (premenopausal or postmenopausal [never, past, or current menopausal hormone use], women only), smoking status (current, former, never), physical activity (quintiles of MET-hours/week), and total energy intake (quintiles of total caloric intake/day). Fixed-effects inverse-variance weighted meta-analysis was used to combine cohort-specific results.
