# Supplementary material for: Polygenic scores, diet quality, and type 2 diabetes risk: An observational study among 35,759 adults from 3 US cohorts
Source: PLoS Med. 2022 Apr 26;19(4):e1003972. doi: 10.1371/journal.pmed.1003972 (PMC9041832; doi:10.1371/journal.pmed.1003972)
Supplement: S4 Table — (DOCX) [file pmed.1003972.s015.docx]

**S4 Table: Correlation between polygenic scores included in this study.**

|  | **Global polygenic score** | **Beta-cell dysfunction** | **Impaired insulin synthesis** | **Obesity-mediated insulin resistance** | **Body fat distribution** | **Lipid/hepatic metabolism** |
| --- | --- | --- | --- | --- | --- | --- |
| **NHS** | | | | | | |
| Global polygenic score | 1 |  |  |  |  |  |
| Beta-cell dysfunction | 0.195 | 1 |  |  |  |  |
| Impaired insulin synthesis | 0.092 | 0.269 | 1 |  |  |  |
| Obesity-mediated insulin resistance | 0.078 | 0.199 | 0.134 | 1 |  |  |
| Body fat distribution | 0.209 | 0.193 | 0.218 | 0.213 | 1 |  |
| Lipid/hepatic metabolism | 0.114 | 0.162 | 0.085 | 0.180 | 0.204 | 1 |
| **HPFS** | | | | | | |
| Global polygenic score | 1 |  |  |  |  |  |
| Beta-cell dysfunction | 0.209 | 1 |  |  |  |  |
| Impaired insulin synthesis | 0.120 | 0.250 | 1 |  |  |  |
| Obesity-mediated insulin resistance | 0.104 | 0.198 | 0.132 | 1 |  |  |
| Body fat distribution | 0.222 | 0.208 | 0.196 | 0.227 | 1 |  |
| Lipid/hepatic metabolism | 0.113 | 0.154 | 0.111 | 0.191 | 0.214 | 1 |
| **NHS II** |  |  |  |  |  |  |
| Global polygenic score | 1 |  |  |  |  |  |
| Beta-cell dysfunction | 0.213 | 1 |  |  |  |  |
| Impaired insulin synthesis | 0.103 | 0.250 | 1 |  |  |  |
| Obesity-mediated insulin resistance | 0.074 | 0.190 | 0.114 | 1 |  |  |
| Body fat distribution | 0.228 | 0.214 | 0.202 | 0.213 | 1 |  |
| Lipid/hepatic metabolism | 0.117 | 0.172 | 0.108 | 0.179 | 0.196 | 1 |

**Table Legend:** Correlations estimated using the Pearson’s correlation test.

Abbreviations: NHS, Nurses’ Health Study; HPFS, Health Professionals Follow-up Study; NHS II, Nurses’ Health Study II
