# Supplementary material for: Polygenic scores, diet quality, and type 2 diabetes risk: An observational study among 35,759 adults from 3 US cohorts
Source: PLoS Med. 2022 Apr 26;19(4):e1003972. doi: 10.1371/journal.pmed.1003972 (PMC9041832; doi:10.1371/journal.pmed.1003972)
Supplement: S5 Table — (DOCX) [file pmed.1003972.s016.docx]

**S5 Table: Associations of global and process-specific polygenic scores with type 2 diabetes risk, random-effects meta-analysis.**

| **Polygenic scores** |  | | |
| --- | --- | --- | --- |
|  | **HR (95%CI)** | ***P* Value** | ***I^2^*** |
| **Global polygenic score** | 1.31 (1.19, 1.44) | <0.001 | 88.5 |
| **Pathway-specific polygenic scores** |  |  |  |
| 1. *Impaired insulin secretion* |  |  |  |
| 1. Beta-cell dysfunction | 1.27 (1.21, 1.33) | <0.001 | 55.5 |
| 2. Impaired insulin synthesis | 1.14 (1.10, 1.17) | <0.001 | 0 |
| 1. *Impaired insulin sensitivity* |  |  |  |
| 1. Obesity-mediated insulin resistance | 1.09 (1.04, 1.14) | <0.001 | 49.1 |
| 2. Body fat distribution | 1.23 (1.19, 1.26) | <0.001 | 0 |
| 3. Lipid/hepatic metabolism | 1.11 (1.07, 1.16) | <0.001 | 0 |

**Table Legend:** For each polygenic score the combined estimates, *P* value, and heterogeneity are shown. Cox proportional hazards models were stratified by age and adjusted for time-varying covariates including ancestry-derived principal components (not time-varying), family history of diabetes (not time-varying), history of hypertension, history of hypercholesterolemia, menopausal status (women only), BMI, smoking status, physical activity, and total energy intake.

Combined estimates from inverse variance-weighted random-effects meta-analysis represent the risk of type 2 diabetes per increment of 1SD in polygenic scores.
