# Supplementary material for: Polygenic scores, diet quality, and type 2 diabetes risk: An observational study among 35,759 adults from 3 US cohorts
Source: PLoS Med. 2022 Apr 26;19(4):e1003972. doi: 10.1371/journal.pmed.1003972 (PMC9041832; doi:10.1371/journal.pmed.1003972)
Supplement: S6 Table — Secondary analyses using the DASH score. DASH, Dietary Approaches to Stop Hypertension. (DOCX) [file pmed.1003972.s017.docx]

**S6 Table: Multiplicative interactions between diet quality and genetic risk using global and pathway-specific polygenic scores. Secondary analyses using the DASH score.**

|  | **Global polygenic score** | **Impaired insulin secretion** | | **Impaired insulin sensitivity** | | |
| --- | --- | --- | --- | --- | --- | --- |
| **Polygenic score** |  | **Beta-cell dysfunction** | **Impaired proinsulin synthesis** | **Obesity-mediated insulin resistance** | **Body fat distribution** | **Lipid/hepatic metabolism** |
| **Multiplicative interactions** | | | | | | |
| Interaction term, coefficient | 0.99 (0.93, 1.06) | 0.98 (0.92, 1.04) | 0.98 (0.92, 1.04) | 1.03 (0.96, 1.10) | 0.95 (0.90, 1.00) | 0.96 (0.91, 1.02) |
| Interaction term, *P* Value | 0.78 | 0.44 | 0.46 | 0.58 | 0.05 | 0.29 |

**Table Legend:** Multivariable-adjusted risk of type 2 diabetes estimated from Cox proportional hazards models stratified by age and adjusted for time-varying covariates including ancestry-derived principal components (not time-varying), family history of diabetes (not time-varying), history of hypertension, history of hypercholesterolemia, menopausal status (women only), BMI, smoking status, physical activity, and total energy intake. Represented are estimates for the cross-product interaction term.
