# Supplementary material for: Polygenic scores, diet quality, and type 2 diabetes risk: An observational study among 35,759 adults from 3 US cohorts
Source: PLoS Med. 2022 Apr 26;19(4):e1003972. doi: 10.1371/journal.pmed.1003972 (PMC9041832; doi:10.1371/journal.pmed.1003972)
Supplement: S7 Table — (DOCX) [file pmed.1003972.s018.docx]

**S7 Table: Additive interactions between diet quality and genetic susceptibility on type 2 diabetes risk, crude models.**

|  | **Global polygenic score** | **Pathway-specific polygenic scores** | | | | |
| --- | --- | --- | --- | --- | --- | --- |
|  |  | **Impaired insulin secretion** | | **Impaired insulin sensitivity** | | |
| **Polygenic score** |  | **Beta, cell dysfunction** | **Impaired proinsulin synthesis** | **Obesity-mediated insulin resistance** | **Body fat distribution** | **Lipid/hepatic metabolism** |
| **Main effects** |  |  |  |  |  |  |
| Diet quality^†^ | 1.50 (1.41, 1.60) | 1.51 (1.42, 1.61) | 1.50 (1.41, 1.59) | 1.49 (1.40, 1.59) | 1.50 (1.41, 1.60) | 1.49 (1.40, 1.59) |
| Polygenic score^‡^ | 1.43 (1.37, 1.50) | 1.31 (1.25, 1.37) | 1.18 (1.12, 1.23) | 1.08 (1.03, 1.14) | 1.28 (1.22, 1.34) | 1.16 (1.10, 1.21) |
| Joint effect | 2.12 (2.01, 2.20) | 1.88 (1.81, 1.95) | 1.68 (1.60, 1.75) | 1.64 (1.57, 1.71) | 1.86 (1.79, 1.93) | 1.65 (1.57, 1.72) |
| **Relative excess risk due to interaction** |  |  |  |  |  |  |
| Relative excess risk due to interaction | 0.19 (-0.01, 0.38) | 0.05 (-0.04, 0.15) | 0.01 (-0.08, 0.10) | 0.07 (-0.02, 0.15) | 0.07 (-0.02, 0.17) | -0.01 (-0.09, 0.08) |
| *P* value | 0.061 | 0.257 | 0.834 | 0.120 | 0.122 | 0.891 |
| **Attributable risk proportion, %** |  |  |  |  |  |  |
| Diet quality | 44.6 (39.7, 49.8) | 58.5 (52.4, 64.5) | 73.1 (65.2, 80.9) | 76.5 (68.2, 84.8) | 58.4 (52.3, 64.6) | 76.7 (68.6, 84.8) |
| Polygenic score | 38.8 (33.8, 43.7) | 35.3 (29.5, 41.1) | 25.6 (18.2, 32.3) | 13.1 (4.5, 21.7) | 32.9 (27, 38.9) | 24.2 (16.4, 32.1) |
| Additive interaction | 16.5 (-0.5, 34.8) | 6.2 (-4.2, 16.1) | 1.4 (-11.4, 14.2) | 10.3 (-1.9, 22.6) | 8.6 (-1.7, 18.9) | -0.9 (-13.9, 12.1) |

**Table Legend:** Risk of type 2 diabetes estimated from Cox proportional hazards stratified by age and adjusted for ancestry-derived principal components.

† Low quality diet vs. high quality diet was defined as a categorical variable based on the median distribution of the diet quality score.

‡ Genetic risk defined using the global polygenic score. Estimated effect sizes per SD increase.
