# Supplementary material for: Polygenic scores, diet quality, and type 2 diabetes risk: An observational study among 35,759 adults from 3 US cohorts
Source: PLoS Med. 2022 Apr 26;19(4):e1003972. doi: 10.1371/journal.pmed.1003972 (PMC9041832; doi:10.1371/journal.pmed.1003972)
Supplement: S8 Table — (DOCX) [file pmed.1003972.s019.docx]

**S8 Table: Additive interactions between diet quality and genetic susceptibility on type 2 diabetes risk in each cohort.**

| **Global polygenic score** | **Nurses’ Health Study** | **Health Professionals Follow-up Study** | **Nurses’ Health Study II** |
| --- | --- | --- | --- |
| **Additive interactions** | | | |
| **Main effects** |  |  |  |
| Diet quality^†^ | 1.22 (1.11, 1.33) | 1.26 (1.11, 1.43) | 1.16 (1.00, 1.35) |
| Polygenic score^‡^ | 1.26 (1.20, 1.31) | 1.23 (1.16, 1.31) | 1.46 (1.37, 1.56) |
| Joint effect | 1.52 (1.42, 1.62) | 1.55 (1.41, 1.70) | 1.68 (1.52, 1.83) |
| **Relative excess risk due to interaction** |  |  |  |
| Relative excess risk due to interaction | 0.03 (-0.09, 0.15) | 0.07 (-0.1, 0.24) | 0.04 (-0.15, 0.22) |
| P Value | 0.62 | 0.41 | 0.69 |
| **Attributable proportion, %** |  |  |  |
| Low diet quality | 41.5 (28.3, 54.7) | 46.7 (29.8, 63.5) | 23.2 (3.6, 42.8) |
| High polygenic score | 52.9 (40, 65.7) | 40.5 (23.4, 57.6) | 71.3 (52.9, 89.6) |
| Additive interaction | 5.6 (-16.1, 27.4) | 12.8 (-15.3, 40.9) | 5.5 (-21.2, 32.2) |

**Table Legend:** Multivariable-adjusted risk of type 2 diabetes estimated from Cox proportional hazards models adjusted stratified by age and adjusted for time-varying covariates including ancestry-derived principal components (not time-varying), family history of diabetes (not time-varying), history of hypertension, history of hypercholesterolemia, menopausal status (women only), BMI, smoking status, physical activity, and total energy intake.

† Low quality diet vs. high quality diet was defined as a categorical variable based on the median distribution of the diet quality score.

‡ Genetic risk defined using the global polygenic score. Estimated effect sizes per SD increase.
