# Supplementary material for: Polygenic scores, diet quality, and type 2 diabetes risk: An observational study among 35,759 adults from 3 US cohorts
Source: PLoS Med. 2022 Apr 26;19(4):e1003972. doi: 10.1371/journal.pmed.1003972 (PMC9041832; doi:10.1371/journal.pmed.1003972)
Supplement: S9 Table — Secondary analyses using the DASH score. DASH, Dietary Approaches to Stop Hypertension. (DOCX) [file pmed.1003972.s020.docx]

**S9 Table: Additive interactions between diet quality and genetic risk using global and pathway-specific polygenic scores. Secondary analyses using the DASH score.**

|  | **Global polygenic score** | **Impaired insulin secretion** | | **Impaired insulin sensitivity** | | |
| --- | --- | --- | --- | --- | --- | --- |
| **Polygenic score** |  | **Beta-cell dysfunction** | **Impaired insulin synthesis** | **Obesity-mediated insulin resistance** | **Body fat distribution** | **Lipid/hepatic metabolism** |
| **Additive interactions** | | | | | | |
| **Main effects** |  |  |  |  |  |  |
| Diet quality^†^ | 1.16 (1.09, 1.24) | 1.17 (1.10, 1.25) | 1.16 (1.09, 1.24) | 1.16 (1.09, 1.24) | 1.18 (1.11, 1.26) | 1.17 (1.09, 1.24) |
| Polygenic score | 1.29 (1.25, 1.33) | 1.26 (1.22, 1.30) | 1.14 (1.10, 1.17) | 1.09 (1.05, 1.12) | 1.23 (1.19, 1.26) | 1.11 (1.07, 1.16) |
| Joint effect | 1.50 (1.43, 1.57) | 1.46 (1.39, 1.54) | 1.33 (1.26, 1.41) | 1.25 (1.18, 1.33) | 1.42 (1.35, 1.49) | 1.27 (1.20, 1.35) |
| **Relative excess risk due to interaction** |  |  |  |  |  |  |
| Relative excess risk due to interaction | 0.04 (-0.05, 0.12) | 0.02 (-0.07, 0.1) | 0.04 (-0.03, 0.13) | -0.01 (-0.08, 0.07) | -0.02 (-0.12, 0.4) | -0.02 (-0.1, 0.05) |
| P Value | 0.40 | 0.78 | 0.19 | 0.86 | 0.35 | 0.56 |
| **Attributable proportion, %** |  |  |  |  |  |  |
| Low diet quality | 32.8 (22.4, 43.2) | 36.9 (26.4, 47.4) | 48.2 (34.8, 61.2) | 64.5 (48.4, 80.5) | 43.4 (32.3, 54.5) | 61.0 (45.9, 76.2) |
| High polygenic score | 60.0 (50.2, 69.9) | 60.6 (50.5, 70.6) | 36.5 (22.3, 50.6) | 38.2 (20.5, 55.9) | 65.9 (55.0, 76.8) | 47.0 (31.3, 62.7) |
| Additive interaction | 7.2 (-8.8, 23.2) | 2.5 (-15.2, 20.2) | 15.8 (-6.1, 36.9) | -2.7 (-31.6, 26.3) | -9.3 (-29.6, 11.0) | -8.1 (-36.0, 20.0) |

**Table Legend:** Multivariable-adjusted risk of type 2 diabetes estimated from Cox proportional hazards models stratified by age and adjusted for time-varying confounders including ancestry-derived principal components (not time-varying), family history of diabetes (not time-varying), history of hypertension, history of hypercholesterolemia, menopausal status (women only), BMI, smoking status, physical activity, and total energy intake.

† Low quality diet vs. high quality diet was defined as a categorical variable based on the median distribution of the diet quality score.
