# Supplementary material for: Polygenic scores, diet quality, and type 2 diabetes risk: An observational study among 35,759 adults from 3 US cohorts
Source: PLoS Med. 2022 Apr 26;19(4):e1003972. doi: 10.1371/journal.pmed.1003972 (PMC9041832; doi:10.1371/journal.pmed.1003972)
Supplement: S10 Table — BMI, body mass index. (DOCX) [file pmed.1003972.s021.docx]

**S10 Table: Interplay between diet quality and pathway-specific polygenic scores on type 2 diabetes risk by changes in BMI.**

|  | **Impaired insulin secretion** | | **Impaired insulin sensitivity** | | |
| --- | --- | --- | --- | --- | --- |
| **Polygenic score** | **Beta-cell dysfunction** | **Impaired insulin synthesis** | **Obesity-mediated insulin resistance** | **Body fat distribution** | **Lipid/hepatic metabolism** |
| **3-way interaction analysis** |  |  |  |  |  |
| Interaction term, *P* Value | 0.74 | 0.51 | 0.08 | 0.80 | 0.80 |

**Table Legend:** Interplay between diet quality and genetic susceptibility assessed using pathway-specific polygenic scores on type 2 diabetes risk based on time-varying BMI (BMI<25kg/m^2^, BMI >25 and <30 kg/m^2^, BMI>30 kg/m^2^). For each polygenic score the combined interaction term P-value is shown. Multivariate analyses were stratified by age and adjusted for time-varying ancestry-derived principal components (not time-varying), family history of diabetes (not time-varying), history of hypertension, history of hypercholesterolemia, menopausal status (women only), smoking status, physical activity, and total energy intake.

Findings suggest that changes in BMI did not modify the risk of type 2 diabetes attributed to increased genetic risk and low diet quality.
