# Supplementary material for: Polygenic scores, diet quality, and type 2 diabetes risk: An observational study among 35,759 adults from 3 US cohorts
Source: PLoS Med. 2022 Apr 26;19(4):e1003972. doi: 10.1371/journal.pmed.1003972 (PMC9041832; doi:10.1371/journal.pmed.1003972)
Supplement: S1 Text — (DOCX) [file pmed.1003972.s022.docx]

**S1 Text: Type 2 diabetes polygenic scores**

To generate a global polygenic score for type 2 diabetes, we initially used data from a random sample of 391,147 participants within UK Biobank with white genetically confirmed racial/ethnic background. We conducted a genome-wide association analysis for type 2 diabetes (n= 17,403 cases) using linear mixed models implemented in BOLT-LMM to account for individual relatedness [1]. Type 2 diabetes cases were defined based on self-reported diagnosis and secondary care data according to ICD-10 code (E10, E11, E13, or E14). Models were adjusted for age, age-square, sex, study center, and the first 20 principal components. Based on the genome-wide summary data, and after excluding variants with minor allele frequency < 1%, we applied the LDPred algorithm, a computational algorithm that calculates a posterior mean effect size for each variant in the genome based on a prior and subsequent linkage disequilibrium shrinkage and a tuning parameter that denotes the proportion of variants with non-zero coefficients [2]. We used a linkage disequilibrium reference panel of 503 European samples from 1000 Genomes phase 3 version 5, ranging causal fractions from 0.001 to 1. In total, ~850,000 independent genetic variants were used to calculate the global polygenic score.

We next tested for the predictive performance of the global polygenic score in an internal validation set within UK Biobank (n= 20,000 participants, 893 type 2 diabetes cases). For everyone in the UK Biobank, the number of associated alleles weighted by the log of the odds ratio was counted and summed across all genetic variants. The polygenic scores were then z standardized. The polygenic score model with a causal fraction of 0.03 archived the highest prediction (AUC=0.72) in this internal validation set and was considered as the optimal model and then applied to our study population (Table A in S1 Text). The predictive capability of the global polygenic score was slightly lower in an analysis in the Nurses’ Health Study (NHS), the Health Professionals’ Follow-up Study (HPFS), and the Nurses’ Health Study II (NHS II) that included all individuals with genetic data (n=6,053 type 2 diabetes cases and n=36,384 controls).

To calculate individual scores in our study population, the number of associated alleles weighted by its relative effect size on type 2 diabetes obtained in UK Biobank was counted and summed across all genetic variants. The scores were then z standardized. We used either directly genotyped or imputed variants with good imputation quality (info > 0.8) and the global polygenic score included the same number of genetic variants across the three cohorts.

| **Casual fraction** | **UK Biobank** | **NHS, HPFS and NHS II** |
| --- | --- | --- |
| ρ=1 | 0.712 | 0.631 |
| ρ=0.3 | 0.714 | 0.633 |
| ρ=0.1 | 0.717 | 0.637 |
| ρ=0.03 | 0.723 | 0.638 |
| ρ=0.01 | 0.698 | 0.610 |
| ρ=0.003 | 0.685 | 0.596 |
| ρ=0.001 | 0.719 | 0.601 |

Note: Data represent AUC calculated under different causal fraction thresholds using logistic regression including the global polygenic score, age, sex and the top 4 PCs. Analysis in the NHS I and II and HPFS included all individuals with genetic data.

To generate pathway-specific polygenic scores for type 2 diabetes based on variants that share increased risk through specific intermediary processes we used summary statistics reported from Udler MS et al [3]. According to this publication, we generated five pathway-specific polygenic scores that are indicative of mechanisms leading to impaired insulin secretion (one polygenic score for beta-cell function and another for impaired proinsulin secretion) and increased insulin resistance (polygenic scores related to obesity, impaired adipocyte differentiation, and lipid/hepatic metabolism). Genetic variants to compute these polygenic scores and their respective weights are detailed in S2 Table. Pathway-specific polygenic scores were generated by multiplying a variant’s genotype dosage by its weight. All the SNPs to generate these pathway polygenic scores were available in our study population.

**References:**

1. Loh P-R, Tucker G, Bulik-Sullivan BK, Vilhjálmsson BJ, Finucane HK, Salem RM, et al. Efficient Bayesian mixed-model analysis increases association power in large cohorts. Nat Genet. 2015;47(3):284–90.

2. Vilhjálmsson BJ, Yang J, Finucane HK, Gusev A, Lindström S, Ripke S, et al. Modeling Linkage Disequilibrium Increases Accuracy of Polygenic Risk Scores. Am J Hum Genet. 2015;97(4):576–92.

3. Udler MS, Kim J, von Grotthuss M, Bonàs-Guarch S, Cole JB, Chiou J, et al. Type 2 diabetes genetic loci informed by multi-trait associations point to disease mechanisms and subtypes: A soft clustering analysis. PLoS Med. 2018;15(9):e1002654.
