# Supplementary material for: Polygenic scores, diet quality, and type 2 diabetes risk: An observational study among 35,759 adults from 3 US cohorts
Source: PLoS Med. 2022 Apr 26;19(4):e1003972. doi: 10.1371/journal.pmed.1003972 (PMC9041832; doi:10.1371/journal.pmed.1003972)
Supplement: S2 Text — (DOCX) [file pmed.1003972.s023.docx]

**S2 Text: Pre-specified analysis plan**

**1. Background**

Lifestyle and genetic factors are key drivers of type 2 diabetes (T2D), a complex disease that is one of the most serious public health challenges of the twenty-first century [1]. Accumulated evidence supports an inverse association between healthy dietary patterns and the risk of T2D [2–7]. In addition, results from randomized clinical trials have showed that a Mediterranean diet supplemented with extra-virgin olive oil or nuts also had a 52% reduction in diabetes incidence compared to a low-fat diet [8]. However, large variability in how people respond to dietary recommendations has been reported [9–11].

The advent of genome-wide polygenic risk scores has been shown to be useful in identifying many more people at risk to develop T2D than do the usual genetic tests, but its clinical utility has not been proven. Therefore, we propose to examine whether adherence to healthy dietary patterns attenuates the impact of the genetic burden on the risk ok T2D.

**2. General methodological considerations**

This analysis will use data collected from the Nurses’ Health Study (NHS), the Health Professionals Follow-up Study (HPFS), and the Nurses’ Health Study II (NHS II). The study baseline will be set at 1986 for the NHS and HPFS, and 1991 for the NHS II, which was when participants first completed a questionnaire on their medical history and lifestyle characteristics.

Main exclusion: The study will be restricted to those with genome-wide genetic data. Participants for genetic determinations were selected to represent a representative sample of the original sample. We will exclude participants diagnosed with type 2 diabetes, cardiovascular disease (including non-fatal myocardial infarction, fatal coronary heart disease, and fatal and non-fatal stroke), or cancer at baseline, those who had an unusual total energy intake at baseline (<800 kcal or >4,200 kcal/day in men and <500 or >3,500 kcal/day in women), and those who completed only the baseline questionnaire.

**2.1 Primary outcome and exposures**

Outcome:

The primary outcome for this analysis is T2D incidence. Incident T2D will be defined as having a fasting glucose ≥7mmol/l (126mg/dl), random plasma glucose ≥11.1mmol/L (200mg/dl), HbA1c ≥6.5 (available cohorts), being on T2D medications or self-reported diagnosis.

Exposures:

Diet quality:

We will use the Alternate Healthy Eating Index score [6] and the Dietary Approaches to Stop Hypertension score [12] to ascertain adherence to healthy dietary patterns. We will calculate cumulative averages of diet quality using the standard methodology to generate these scores and implemented in the cohorts before.

Type 2 diabetes polygenic scores:

We will build a genome-wide polygenic risk score for T2D used the recently developed LDPred computational algorithm. This involves a Bayesian approach to calculate a posterior mean effect for all variants based on a prior (effect size in the prior GWAS) and subsequent shrinkage based on linkage disequilibrium. We will use UK Biobank as a training set and a linkage disequilibrium reference panel of 503 European samples from 1000 Genomes phase 3 version 5.

In addition, we will generate pathway-specific polygenic scores using genetic variants and weights data from a previous study aimed at grouping known type 2 diabetes loci based on shared physiological similarities.

To predict generate these scores in the cohorts, we will use genotyped variants from each participating cohort with call rate higher than 0.95 and Hardy-Weinberg equilibrium p-value higher than 1×10^-4^. When not directly genotyped, we will use imputed variant based on the criteria of imputation quality (INFO) > 0.7 and MACH r^2^>0.8. If variants included in the provided SNP lists are missing in specific participating cohorts, we will use proxy variants based on available variants reaching r^2^>0.8 with the variant included in the original list.

- 1. **Covariates**

Models will be adjusted for 1) age (in months, continuous) and ancestry-derived principal components (1 to 4), family history of diabetes (yes or no), history of hypertension (yes or no), history of hypercholesterolemia (yes or no), menopausal status (premenopausal or postmenopausal [never, past, or current menopausal hormone use], women only), BMI (quintiles of kg/m), smoking status (current, former, never), physical activity (quintiles of MET-hours/week), and total energy intake (quintiles of total caloric intake/day).

**2.3 Unit of analysis**

We will model diet quality and genetic risk on the continuous scale, and hazard ratios and 95% confidence intervals for type 2 diabetes will be reported per 1SD change in main exposures of interest.

**3. Statistical analyses**

Prior to implementing the analysis, each cohort will provide basic descriptive statistics (mean (SD), min, max) of main exposures and outcome of interest.

Cox proportional-hazard models with repeated measures analyses will be used to calculate hazard ratios and 95% confidence intervals for type 2 diabetes after adjusting for potential confounders. Model 1 will be adjusted for age and principal components. Model 2 will be further adjusted for family history of diabetes, history of hypertension, history of hypercholesterolemia, menopausal status, BMI, smoking status, physical activity, and total energy intake.

We will investigate the presence of additive and multiplicative interactions between diet quality and genetic risk on type 2 diabetes risk. In addition, we will cross-classify participants according to categories of genetic risk and diet quality (nine categories based on thirds of genetic risk and diet quality score) and conducted analyses stratified by genetic risk category.

All analyses will be performed separately for each cohort and then pooled with the use of inverse variance weighted, fixed-effects meta-analysis.

In a sensitivity analysis we will run models without adjusting for BMI and combine cohort-level estimates using random-effects meta-analysis.

**References**

1. Abajobir AA, Abate KH, Abbafati C, Abbas KM, Abd-Allah F, Abdulle AM, et al. Global, regional, and national comparative risk assessment of 84 behavioural, environmental and occupational, and metabolic risks or clusters of risks, 1990–2016: a systematic analysis for the Global Burden of Disease Study 2016. Lancet. 2017;390(10100):1345–22.

2. Martinez-Gonzalez MA, de la Fuente-Arrillaga C, Nunez-Cordoba JM, Basterra-Gortari FJ, Beunza JJ, Vazquez Z, et al. Adherence to Mediterranean diet and risk of developing diabetes: prospective cohort study. BMJ. 2008;336(7657):1348–51.

3. Liese AD, Nichols M, Sun X, D’Agostino RB, Haffner SM. Adherence to the DASH Diet Is Inversely Associated with Incidence of Type 2 Diabetes: The Insulin Resistance Atherosclerosis Study. Diabetes Care. 2009;32(8)1434–6.

4. de Koning L, Chiuve SE, Fung TT, Willett WC, Rimm EB, Hu FB. Diet-Quality Scores and the Risk of Type 2 Diabetes in Men. Diabetes Care. 2011;34(5):1150–6.

5. InterAct Consortium, Romaguera D, Guevara M, Norat T, Langenberg C, Forouhi NG, et al. Mediterranean Diet and Type 2 Diabetes Risk in the European Prospective Investigation into Cancer and Nutrition (EPIC) Study: The InterAct project. Diabetes Care. 2011;34(9):1913–8.

6. Chiuve SE, Fung TT, Rimm EB, Hu FB, McCullough ML, Wang M, et al. Alternative dietary indices both strongly predict risk of chronic disease. J Nutr. 2012;142(6):1009–18.

7. InterAct Consortium TI. Adherence to predefined dietary patterns and incident type 2 diabetes in European populations: EPIC-InterAct Study. Diabetologia. 2014;57(2):321–33.

8. Salas-Salvadó J, Bulló M, Estruch R, Ros E, Covas M-I, Ibarrola-Jurado N, et al. Prevention of diabetes with Mediterranean diets: a subgroup analysis of a randomized trial. Ann Intern Med. 2014;160(1):1–10.

9. Evert AB, Dennison M, Gardner CD, Garvey WT, Lau KHK, MacLeod J, et al. Nutrition Therapy for Adults with Diabetes or Prediabetes: A Consensus Report. Diabetes care. 2019;42(5):731–54.

10. Fumagalli M, Moltke I, Grarup N, Racimo F, Bjerregaard P, Jorgensen ME, et al. Greenlandic Inuit show genetic signatures of diet and climate adaptation. Science. 2015;349(6254):1343–7.

11. Goni L, Qi L, Cuervo M, Milagro FI, Saris WH, MacDonald IA, et al. Effect of the interaction between diet composition and the *PPM1K* genetic variant on insulin resistance and β cell function markers during weight loss: results from the Nutrient Gene Interactions in Human Obesity: implications for dietary guidelines. Am J Clin Nutr. 2017;106(3):902–8.

12. Fung TT, Chiuve SE, McCullough ML, Rexrode KM, Logroscino G, Hu FB. Adherence to a DASH-style diet and risk of coronary heart disease and stroke in women. Arch Intern Med. 2008;168(7):713–20.
