# Supplementary material for: Polygenic scores, diet quality, and type 2 diabetes risk: An observational study among 35,759 adults from 3 US cohorts
Source: PLoS Med. 2022 Apr 26;19(4):e1003972. doi: 10.1371/journal.pmed.1003972 (PMC9041832; doi:10.1371/journal.pmed.1003972)
Supplement: Attachment — Submitted filename: Comments_PRS_Diet_T2D_PLoSMed_15July2021_clean.docx [file pmed.1003972.s025.docx]

**Comments on the manuscript “Polygenic scores, diet quality, and type 2 diabetes risk: a prospective, observational study” (PMEDICINE-D-21-01465R1)**

Merino et al examined the joint effects of diet quality and genetic risk on incident T2D in three large prospective cohorts followed up for 902,386 person-years with available genetic data. This is a timely topic given the emerging concept of precision prevention with the implication that individuals with high genetic risk may benefit more from intervention. The authors leveraged the comprehensive genotype and phenotype data in UK Biobank to construct and validate a global polygenic score, as well as several pathway-specific polygenic scores for T2D, which were then analyzed jointly with the diet quality scores quantified by two different methods. The authors also conducted a three-way interaction analysis to investigate whether changes in BMI could modify the joint association of genetic risk and diet quality with T2D. The authors confirmed the independent associations of incident T2D with diet quality and genetic risk albeit without significant interaction and was not modified by BMI. They also quantified that 50% of the proportion of diabetes risk was explained by genetic risk and 38% by high quality diet. Within the polygenic score, ~60% was attributable to beta-cell dysfunction, although sub-analysis showed similar joint associations of quality diet and different pathway-specific polygenic risk scores. Overall, this is a well-conducted study in terms of design, sample size, cohort selection, exposure and outcome definition, and sensitivity analyses although some issues to be addressed.

Major issues:

One of the major limitations of this study is the applicability of the results to the general population since the majority of these subjects were well informed and educated healthcare professionals and predominantly nurses. Given the importance of education on diabetes risk, this point had not been discussed. In table 1, the author should state specifically the single sex nature in the 3 cohorts including 35,759 men and women. Although the authors concluded that high quality diet was associated with reduced risk of T2D irrespective of genetic burden, they proposed the use of polygenic score for surveillance purpose but did not discuss the implication of high quality diet within this context.

*Background*

1) Page 4, line 71-74: the authors listed four limitations in existing studies that prevented the researchers from identifying “genotypes interacting with lifestyle or dietary exposures” but did not address them in their analysis or discussion.

- Limitation 1: *Partial characterization of genetic risk due to a limited number of variants*. There are other genetic loci associated with other traits that may interact with diet or lifestyle on T2D genetic risk (e.g. <https://pubmed.ncbi.nlm.nih.gov/33864366/> reported gene-diet interactions that influenced HbA1c ). The “partial characterization of genetic risk” is not only restricted to “limited number of variants” but rather the specificity, sensitivity and effect size of these scores. In Figure 1, the “Beta-cell dysfunction” score constructed from fewer of SNPs compared with the “850,000 independent variants” used for the global polygenic score, yielded comparable hazard ratios on incident T2D. Thus, increasing the number of T2D risk loci does not necessarily provide additional variance in discriminating subjects with different T2D genetic risk. In the Appendix, the global polygenic score for T2D together with sex and age had an AUC of 0.638, what is the AUC when they are combined with the dietary scores in explaining the total variance? Given the differences in genetic architecture and many non-genetic factors across various populations and settings, the continuing focus of using aggregated polygenic risk score to detect gene-environment interaction continues to impede our understanding in the effects of environment-personal-lifestyle-familial factors on the development of T2D.
- Limitation 2: *Interactions in previous studies were predominantly assessed on the multiplicative scale alone*. The authors only presented the additive Relative Excess Risk due to Interaction (RERI) in Table 2. The authors should include the index estimates with confidence intervals and *P* values of multiplicative interactions for completeness.
- Limitation 3: *Single time point dietary exposure*. The cohorts had multiple diet quality scores collected at different time points. The authors should state clearly i) whether the diet quality scores were used as a time-fixed variable or a time-varying variable in the Cox model; ii) compared with the single time point diet quality score, e.g. the last one before endpoint, what was the advantage of the cumulative average? Was the cumulative average sufficient to capture the longitudinal trajectory of the score?
- Limitation 4: *Limited follow-up*. The three cohorts were followed up from 26 years to 30 years. With such long follow-up, considerable changes in the socioeconomic and demographic characteristics and ecological factors could influence greatly on diet quality and T2D incidence. Did the author adjust for any socioeconomic covariates in their Cox regression analyses if there were relevant information in the questionnaire, or discuss the issue if not available. Did they adjust for year of data capture?

*Methods*

2) For the global polygenic risk scores and pathway-specific risk scores, suggest

- present the distribution of these PRS in the three cohorts separately;
- show overlap between the SNPs in the global and pathway-specific PRS;
- show correlation among the global PRS and pathway-specific PRSs
- discuss AUC differences between UK Biobank validation dataset (0.723) and combined cohort under study (0.638)

3) Regarding the diet quality assessed at baseline and every 4 years thereafter,

- What was the average number of diet quality scores for a subject in the three cohorts respectively?
- Was there any correlation between the number of diet quality scores and the cumulative average of the scores (i.e. whether the subjects with more diet quality scores available tended to have a higher cumulative average)? If so, this may be a marker of adherence and would that affect the interaction?
- What was the within-subject variance distribution in the three cohorts respectively? Is the cumulative average a valid surrogate of the long-term diet trajectory for subjects especially in those with fluctuating diet quality and large within-subject variance.
- Was there any difference in the within-subject variance among the three cohorts?
- The authors stopped updating dietary information when the subjects first reported having cardiovascular events or cancers. What about other factors that could dramatically change subjects’ diet habits (e.g. T2D education or elevated glycemic traits that the subject may be aware of)?
- Did family history (FH) of diabetes affect the diet quality scores? Genetics is only a component of FH, did the authors repeat the analysis in those with or without FH?

4) Page 6, line 183: The statement “Models were adjusted for time-varying …” is very vague. Please define the time-varying covariates and the “a priori” rationale for considering them as time-varying risk factors. Similarly, what were the time-fixed covariates in the models and the values at which time-point were used. A conceptual framework including these mediators or confounders would be helpful in explaining the selection of these covariates in the model.
5) Page 6, line 185: The statement “The fully adjusted model was further adjusted …” is contradictory.
6) Page 6, line 190-191: “Because time varying BMI could be on the causal pathway between diet quality and …, we also conducted separate models without adjusting for BMI”. Where were the results of these separate models? In supplementary figure 7, the results are presented in 3 categories of BMI, was this the baseline BMI rater than changes in BMI between baseline and censor point?
7) Page 10, line 200: What was the “median **distribution** of the diet quality score in each cohort”? The statement is confusing from a statistical aspect.
8) Page 10, line 200: The RERI could also be calculated on a multiplicative scale to provide the index estimates of multiplicative interaction.
9) Page 10, line 205: “tested for multiplicative interactions by comparing the -2 log likelihood”. The statement “-2 log likelihood” was informal. In addition, what test was used?
10) Page 10, line 209: The statement “examine whether changes in BMI modified the joint association …” is also vague. Which one was used to perform the 3-way interaction, time varying BMI or changes between two BMI?

*Results*

11) In Table 1, please include the incidence of T2D, incidence of death, number of lost to follow up and number of cardiovascular/cancer events in the three cohorts.
12) Page 12, line 246: “there was a risk gradient with increasing …”. Please perform trend analysis to demonstrate the statistical significance of such “risk gradient”.
13) Page 12, line 251-252: “The available sample gave us 80% statistical power …”, at what significance level?
14) Given that the three cohorts were all sex-specific (two women cohorts and one men cohort), did the authors observe any sex-specific association between the risk factors under study and T2D incidence?
15) The lack of interactions between T2D genetic risk scores and diet quality on incident T2D limited the novelty of this work. A recent study reported interaction between fruit intake and T2D genetic risk (<https://pubmed.ncbi.nlm.nih.gov/33399975/>). Did the author explore the association of a subset of foods or nutrients well known for their risk-conferring or risk-reducing effect for T2D instead of using the aggregated score .
16) Have the authors performed GWAS on dietary traits or borrowed similar information from the UK Biobank analysis (<https://pubmed.ncbi.nlm.nih.gov/32193382/>) to investigate whether there are shared genetic factors between T2D and unhealthy diet. Other similar work such as genome-wide gene-diet interaction analysis should preferably be performed or cited to reflect the limitation of the study (<https://pubmed.ncbi.nlm.nih.gov/33864366/>). These analyses would complement the existing results and strengthen this study.
17) Limitations and strengths of the study should be discussed.

Minor issues:

1) Page 4, line 82: “processes such impaired insulin …” 🡺 “processes such **as** impaired insulin …”
2) Page 6, line 116: “… a global polygenic score for type 2 diabetes that capture overall …” 🡺 “that capture**s** overall …”
3) Page 7, line 138: “transcriptional regulation (13)’ indicating …” 🡺 “transcriptional regulation (13)**,** indicating …”
4) Page 7, line 144-145: “in this study with different the number of …” 🡺 “in this study with different number of”
5) Page 7, line 154: “we used the DASH score (Dietary Approaches to Stop Hypertension) …” 🡺 “we used the **Dietary Approaches to Stop Hypertension (DASH)** score …”
6) Page 10, line 202: “which is the proportion of risk duet to …” 🡺 “… **due** to …”
7) Page 26, line 566: “… or median (IQR) …” 🡺 “… or median**s** (IQR) …”
8) Page 27, line 577: “time varying confounders including age, ancestry-derived principal components, family history …”. How could the genetic principal components be used as time varying covariates? As for family history, can change in status over time influence dietary habit and thence risk of diabetes.
