# Supplementary material for: Polygenic scores, diet quality, and type 2 diabetes risk: An observational study among 35,759 adults from 3 US cohorts
Source: PLoS Med. 2022 Apr 26;19(4):e1003972. doi: 10.1371/journal.pmed.1003972 (PMC9041832; doi:10.1371/journal.pmed.1003972)
Supplement: Attachment — Submitted filename: Response.PMEDICINE-D-21-01465R1.docx [file pmed.1003972.s026.docx]

**October 5, 2021**

**Caitlin Moyer, Ph.D.**

**Associate Editor, *PLoS Medicine***

**Ref: PMEDICINE-D-21-01465R1**

Dear Dr. Moyer:

Please find attached a revised version of our manuscript, entitled “**Polygenic scores, diet quality, and type 2 diabetes risk: a prospective, observational study**” for continued consideration as an original investigation in *PLoS Medicine*.

We thank the reviewers for their positive and thoughtful comments, which we believe have strengthened the content and presentation of our manuscript. We are grateful for the opportunity to revise our manuscript and respond to you and our peers. In this revised version, we address all comments and concerns raised by the reviewers on a point-by-point basis as detailed below. In the manuscript, we have also highlight the changes in red font.

We look forward to your further review of our manuscript.

Sincerely,

Jordi Merino, Ph.D.

**EDITOR COMMENTS:**

**1. From the Academic Editor: Please emphasize the novelty, clinical significance and interpretation of the findings of the study, as compared to the existing literature describing relationships and interactions between lifestyle and genetic risk and risk of type 2 diabetes. Please provide some discussion on the new methods used in this study.**

Response: We thank the Academic Editor for the favorable view of our research and for the helpful suggestions to improve the manuscript.

The novel aspects of this study include the use of polygenic scores that capture overall type 2 diabetes risk and distinct pathophysiological processes, the systematically evaluation of additive and multiplicative interactions between diet quality and genetic risk on the development of type 2 diabetes, and the use of repeated measurements of diet across the life course.

Our study adds to knowledge on the interplay between genetic and lifestyle factors by showing that both diet quality and genetic risk are independently associated with risk of type 2 diabetes without evidence of additive or multiplicative interactions. We documented that within any genetic risk category, high diet quality compared to low diet quality, was associated with a nearly 30% lower risk of type 2 diabetes. Further, we showed that the risk of type 2 diabetes attributed to the combination of increased genetic risk and low diet quality was similar to the sum of the risks associated which each factor alone.

In the era of precision medicine / nutrition, in which genetics and other axes of biological information are used to predict short-term variable responses to specific foods or diets, our results support public health efforts that emphasize population-wide healthy dietary pattern recommendations for the prevention of type 2 diabetes as genetic risk does not seem to modify their effectiveness. Further, our results emphasize the value of genetic risk assessment to identify individuals at increased disease risk and their potential for risk stratification and surveillance, as those at increased genetic risk might need to adopt other lifestyle behaviors in addition to diet to mitigate their inherited risk.

We have added the following information:

Page 20, line 370: *Our study adds to knowledge on the interplay between genetic and lifestyle factors by formally investigating whether polygenic scores for type 2 diabetes capturing overall genetic risk or distinct pathophysiological mechanisms could help prioritize individuals who would benefit the most from targeted dietary recommendations*

Page 20, line 381: *[…] our study supports that both diet quality and overall or pathway-specific genetic risk are independently associated with risk of type 2 diabetes. These findings suggest that healthy dietary recommendations for the prevention of type 2 diabetes could be deployed across all levels of genetic risk in the population as genetic risk does not seem to modify their effectiveness. Further, our results emphasize the value of genetic risk assessment to identify individuals at increased disease risk and their potential for risk stratification and surveillance, as those at increased genetic risk might need to incorporate other lifestyle components in addition to healthy diet to mitigate their inherited risk.*

Page 21, line 390: *We systematically evaluated the presence of additive and multiplicative interactions between genetic risk and diet quality on type 2 diabetes incidence […] Our findings provide evidence that there is no departure from the additivity of risks attributed to each factor separately, indicating that the presence of the two exposures (low diet and increased genetic risk) does not explain a higher number of cases that could have prevented if only one of the exposures were present*

Page 21, line 404: *By clarifying that genetic risk and diet quality are each independently associated with the risk of type 2 diabetes and would not have an additive or multiplicative impact on the risk of the disease, our findings can yield useful clinical and public health answers as we prepare for the eventual implementation of precision nutrition.*

**2. Data Availability statement: PLOS Medicine requires that the de-identified data underlying the specific results in a published article be made available, without restrictions on access, in a public repository or as Supporting Information at the time of article publication, provided it is legal and ethical to do so. The Data Availability Statement (DAS) requires revision. For each data source used in your study:**

**a) If the data are freely or publicly available, note this and state the location of the data: within the paper, in Supporting Information files, or in a public repository (include the DOI or accession number).**

**b) If the data are owned by a third party but freely available upon request, please note this and state the owner of the data set and contact information for data requests (web or email address). Note that a study author cannot be the contact person for the data.**

**c) If the data are not freely available, please describe briefly the ethical, legal, or contractual restriction that prevents you from sharing it. Please also include an appropriate contact (web or email address) for inquiries (again, this cannot be a study author).**

Response: Thank you for guiding us on this. We have added the following information:

Page 25, line 477: *Data Availability: Information including the procedures to obtain and access data from the Nurses’ Health Study and Health Professionals Follow-Up Study is described at* [*http://www.nurseshealthstudy.org/researchers*](http://www.nurseshealthstudy.org/researchers) *for the Nurses’ Health Study (contact:* [*nhsaccess@channing.harvard.edu*](mailto:nhsaccess@channing.harvard.edu)*) or* [*https://www.hsph.harvard.edu/hpfs/hpfs_collaborators.htm*](https://www.hsph.harvard.edu/hpfs/hpfs_collaborators.htm) *for the Health Professionals Follow-up Study (contact:* [*hpfs@hsph.harvard.edu*](mailto:hpfs@hsph.harvard.edu)*). Access to statistical codes and datasets will be facilitated following the existing data sharing guidelines provided, which can be found on the study websites.*

**3. Abstract: Please structure your abstract using the PLOS Medicine headings (Background, Methods and Findings, Conclusions).**

**4. Abstract Background: Provide the context of why the study is important. The final sentence should clearly state the study question.**

**5. Abstract: Methods and Findings: Please include some summary demographics of the cohorts, and specify the cohorts are US-based.. Please quantify the main results (with 95% CIs and p values). Please include the important dependent variables that are adjusted for in the analyses.**

**6. Abstract: Methods and Findings: In the last sentence of the Abstract Methods and Findings section, please describe the main limitation(s) of the study's methodology.**

Response: Thank you for guiding us on this. We have clarified all these points in the abstract.

Abstract

**Background:** Both genetic and lifestyle factors contribute to the risk of type 2 diabetes, but the extent to which there is a synergistic effect of the two exposures is unclear. The aim of this study was to prospectively examine the joint associations of genetic risk and diet quality with incident type 2 diabetes.

**Methods and Findings:** We analyzed data from 35,759 men and women in the United States participating in the Nurses’ Health Study I (1986-2016) and II (1991-2017) and the Health’s Professional Follow-up Study (1986-2016) with available genetic data, and who did not have diabetes, cardiovascular disease, or cancer at baseline. Genetic risk was characterized using both a global polygenic score capturing overall genetic risk and pathway-specific polygenic scores denoting distinct pathophysiological mechanisms. Diet quality was assessed using the Alternate Healthy Eating Index (AHEI). Cox models were used to calculate hazard ratios for type 2 diabetes after adjusting for potential confounders. Over 902,386 person-years of follow-up, 4,433 participants were diagnosed with type 2 diabetes. The relative risk of type 2 diabetes was 1.29 (95% CI 1.25, 1.32; *P* < 0.001) per SD increase in global polygenic score and 1.13 (1.09, 1.17; *P* < 0.001) per 10-unit decrease in AHEI. Irrespective of genetic risk, low diet quality, as compared to high diet quality, was associated with ~30% increased risk of type 2 diabetes (*P*_interaction_ =0.69). The joint association of low diet quality and increased genetic risk was similar to the sum of the risk associated which each factor alone (*P*_interaction_=0.30). Limitations of this study include the self-report of diet information or the inclusion of highly educated participants with available genetic data.

**Conclusions:** These data provide evidence for the independent associations of genetic risk and diet quality with incident type 2 diabetes and suggest that a healthy diet is associated with lower diabetes risk across all levels of genetic risk.

**7. Author summary: At this stage, we ask that you include a short, non-technical Author Summary of your research to make findings accessible to a wide audience that includes both scientists and non-scientists. The Author Summary should immediately follow the Abstract in your revised manuscript. This text is subject to editorial change and should be distinct from the scientific abstract.**

Response: The Author Summary is now included after the abstract and detailed below:

Author summary

***Why was this study done?***

- *Both genetic and lifestyle factors contribute to individual-level risk of type 2 diabetes.*
- *While previous studies have shown that adherence to healthy diets is associated with reduced type 2 diabetes incidence regardless of genetic risk, the partial characterization of genetic risk and the predominanly assessment of interactions on the multiplicative scale might have prevented previous studies from identifying genetic profiles interacting with dietary exposures.*
- *Therefore, understanding how genetic risk and diet quality contribute to the development of type 2 diabetes is important to support evidence-based preventive interventions.*

***What did the researchers do and find?***

- *In three cohort studies involving 35,759 men and women in the United States, we used novel polygenic scores for type 2 diabetes to systematically evaluate the presence of additive and multiplicative interactions between genetic risk and diet quality on the development of type 2 diabetes.*
- *We found that both low diet quality and increased overall or pathway-specific genetic risk were independently associated with higher risk of type 2 diabetes.*
- *We documented that the risk of type 2 diabetes attributed to the combination of increased genetic risk and low diet quality was similar to the sum of the risks associated which each factor alone*
- *Further, we showed that within any genetic risk category, high diet quality compared to low diet quality, was associated with a nearly 30% lower risk of type 2 diabetes*

***What do these findings mean?***

- *Results from this study suggest that consuming a healthier diet is associated with a lower risk of type 2 diabetes regardless of genetic risk.*
- *Our results underscore the value of genetic risk assessment to identify individuals at increased disease risk and their potential for risk stratification and surveillance.*
- *Such knowledge can serve to inform and design future strategies to advance the prevention of type 2 diabetes.*

**8. Throughout: Please place in-text reference citations within square brackets.**

Response: We have used the appropriate citation style

**9. Methods: Please include the information presented in the Appendix (Description of the study population and sample selection) where the Study design and Population are described.**

Response: Thank you for this suggestion. We have edited the methods section accordingly.

Page 8, line 117: *We used data collected from three prospective cohort studies in the United States including participants in the Nurses’ Health Study (NHS), the Health Professionals’ Follow-up Study (HPFS), and the Nurses’ Health Study II (NHS II) [14,15]. The Nurses’ Health Study (NHS) was established in 1976 when 121,700 female registered nurses aged 30-55 were recruited [14]. The Health Professionals Follow-Up Study (HPFS) began in 1986 and enrolled 51,529 male health professionals aged 40-75 years [15]. The NHS II cohort was initiated in 1989 and included 116,340 women aged 25-42 years [14]. The study baseline was set at 1986 for the NHS and HPFS, and 1991 for the NHS II, which was when participants first completed a questionnaire on their medical history, diet, and lifestyle characteristics.*

*Multiple genome-wide association studies (GWAS) have been conducted within the NHS, NHS II and HPFS nested-cohorts to investigate genetic susceptibility to 12 complex diseases [16]. Participants for genetic determinations were selected to represent a representative sample of the original sample. Demographic characteristics and health status of participants with genetic information were generally similar to those who did not, therefore bias due to selection are probably minimized. Genotype, imputation and quality control of genome-wide genetic data has been harmonized across nested-cohorts and detailed elsewhere [16]. After quality-control, genome-wide genetic data were available for 42,437 individuals. We excluded participants diagnosed with diagnosis of type 2 diabetes (n=3,200), cardiovascular disease (including non-fatal myocardial infarction, fatal coronary heart disease, and fatal and non-fatal stroke, n=613), or cancer at baseline (n=721), those who had an unusual total energy intake at baseline (<800 kcal or >4,200 kcal/day in men and <500 or >3,500 kcal/day in women, n=581), and those who completed only the baseline questionnaire (n=1,563). After these exclusions, 14,454 participants in the NHS, 9,417 participants in the HPFS, and 11,888 participants in the NHS II of predominantly white race/ethnicity were included in this analysis. The study protocol was approved by the human research committee of Brigham and Women's Hospital and the Harvard TH Chan School of Public Health.*

**10. Methods: Line 108: Please change the superscript reference to [10].**

Response: Thank you for this suggestion.

**11. Methods: Diet quality: Please mention how the DASH score was determined. Please mention the food components for the AHEI and DASH.**

Response: We have included this information as suggested.

Page 11, line 193: *To quantify overall diet quality, we calculated the Alternate Healthy Eating Index (AHEI) using food components and scoring criteria that have been described previously [24]. The AHEI score is based on 11 foods and nutrients predictive of chronic disease risk, emphasizing higher intake of vegetables (excluding potatoes), fruits, whole grains, nuts and legumes, long chain (n-3) fatty acids, and polyunsaturated fatty acids; moderate intake of alcohol; and lower intake of sugar sweetened drinks and fruit juice, red and processed meats, trans fat, and sodium. Each component was scored from 0 (unhealthiest) to 10 (healthiest) points, with intermediate values scored proportionally. All component scores were summed to obtain a total score ranging from 0 (non-adherence) to 110 (best adherence) points.*

*As an additional method to quantify diet quality we used the Dietary Approaches to Stop Hypertension (DASH) score [25]. The DASH score was based on eight foods and nutrients that were either emphasized or deemphasized in the DASH-style diet. Each component was scored from 1 to 5 points according to fifths of intake, with 5 being the best score for higher intake of vegetables, fruits, nuts and legumes, whole grains, and low-fat dairy products and for lower intake of sugar sweetened drinks, red and processed meats, and sodium. The total score ranged from eight to 40 points, with higher scores indicating a higher quality diet.*

**12. Methods: Line 162: Please note where a complete list of the covariates/ risk factors may be found.**

Response: Thank you for this suggestion. We have detailed this information in the methods section and in the appropriate figures and tables.

Page 12, line 210: *[…] including age, family history of diabetes, history of hypertension, history of hypercholesterolemia, body* *mass index, menopausal status and postmenopausal hormone use in women, smoking status, physical activity, total energy intake, and alcohol intake.*

**13. Methods: Line 202: Please change “duet” to “due” here.**

Response: Thank you for this suggestion.

**14. Methods: Please ensure that the study is reported according to the STROBE guideline, and include the completed STROBE checklist as Supporting Information. Please add the following statement, or similar, to the Methods: "This study is reported as per the Strengthening the Reporting of Observational Studies in Epidemiology (STROBE) guideline (S1 Checklist)." When completing the checklist, please use section and paragraph numbers, rather than page numbers.**

Response: We confirm our study is reported according to the STROBE guideline and included the STROBE checklist as Supporting information. We have added the following information

Page 15, line 282: *This manuscript is reported as per the Strengthening the Reporting of Observational Studies in Epidemiology (STROBE) guideline (S1 STROBE Checklist) [30].*

**15. Methods: Did your study have a prospective protocol or analysis plan? Please state this (either way) early in the Methods section.**

**a) If a prospective analysis plan (from your funding proposal, IRB or other ethics committee submission, study protocol, or other planning document written before analyzing the data) was used in designing the study, please include the relevant prospectively written document with your revised manuscript as a Supporting Information file to be published alongside your study, and cite it in the Methods section. A legend for this file should be included at the end of your manuscript.**

**b) If no such document exists, please make sure that the Methods section transparently describes when analyses were planned, and when/why any data-driven changes to analyses took place.**

**c) In either case, changes in the analysis-- including those made in response to peer review comments-- should be identified as such in the Methods section of the paper, with rationale.**

Response: We elaborated a pre-specific protocol including information, such as definitions of exposures, outcomes, and covariates, and statistical analysis plan prior to data analysis. The protocol was presented, discussed, and approved in a meeting with study investigators of NHS/NHSII and HPFS prior to data analysis. The protocol has been included in the supplement with the enclosed revision.

Page 12, line 220: *We elaborated a prespecified protocol including definitions of exposures, outcomes and covariates, and statistical analysis plan prior to data analysis (S2 Appendix).*

**16. Results: In the first paragraph, please report on some of the participant characteristics presented in Table 1.**

Response: We have added the following information

Page 16, line 288: *The mean baseline age of NHS participants was 53 years old, 54 in HPFS, and 37 in NHSII. Most of them were of European descent without major chronic diseases at baseline. Mean BMI ranged from 24.3 kg/m^2^ in NHS to 25.5 kg/m^2^ in NHSII. At baseline, mean AHEI score ranged from 48.9 in NHSII to 52.6 in HPFS.*

**17. Results: Please provide 95% CIs and p values for all the main analyses described in the text (e.g PRS association with diabetes risk). For adusted analyses, please note the factors adjusted for (in the text or the appropriate figure/table), and please also provide results from unadjusted analyses.**

Response: Thank you for this suggestion. We have provided 95% CIs and p-values for all analyses, detailed the factors that we adjusted for in the methods section and in the appropriate figures and tables, and provided results from unadjusted analyses in the supplement (Please see below results from unadjusted results in response to questions 25 and 26).

**18. Results: Line 271: Please mention the confounders adjusted for in this analysis.**

Response: We have clarified this point in the methods section.

Page 13, line 242: *Cox regression models were adjusted for age (in months, continuous) and ancestry-derived principal components (1 to 4) (age-adjusted model). The multivariable adjusted model was further adjusted for family history of diabetes (yes or no), history of hypertension (yes or no), history of hypercholesterolemia (yes or no), menopausal status (premenopausal or postmenopausal [never, past, or current menopausal hormone use], women only), BMI (quintiles of kg/m^2^), smoking status (current, former, never), physical activity (quintiles of MET-hours/week), and total energy intake (quintiles of total caloric intake/day).*

**19. Results: Line 275: Please clarify this sentence, as it seems as if there was no evidence for an interaction “...indicating a non-significant interaction on the multiplicative scale (P =0.65 for interaction…)”**

Response: Thank you for this suggestion. We have edited the sentence.

Page 18, line 333: *There was no evidence of significant interactions on the multiplicative scale between diet quality and genetic risk on the risk of type 2 diabetes (P_interaction_ =0.65; Table 3).*

**20. Discussion: Line 288: Please rename this section “Discussion”**

Response: Thanks

**21. Discussion: Please present and organize the Discussion as follows: a short, clear summary of the article's findings; what the study adds to existing research and where and why the results may differ from previous research; strengths and limitations of the study; implications and next steps for research, clinical practice, and/or public policy; one-paragraph conclusion.**

Response. Thank you for guiding us on this. We have re-written the full discussion following your suggestions.

**22. Please remove “Funding” and “Duality of Interests” sections from the main text. Please be sure that all information is completely and accurately entered in the Financial Disclosures and Competing Interests sections of the manuscript submission metadata.**

Response: We have removed these two sections for the main manuscript.

**23. References: Please use the "Vancouver" style for reference formatting, and see our website for other reference guidelines. Please check journal title abbreviations- for example, please check if this should be J Womens Health for reference 15. Please update reference 31. Please use the same formatting for the references in the Appendix.**

Response: We confirm we used the Vancouver style for reference formatting.

**24. Figures and Tables: Please fully define all abbreviations used within each figure and table in the legend, including for the Supporting Information tables.**

Response: We have added definitions for all abreviations used in figures and tables.

**25. Figures 1 and 2: Please provide results from unadjusted analyses in addition to the adjusted analyses.**

Response: Thank you for this suggestion. We have placed age-adjusted estimates for Fig 1 analyses in S3 Table. This table also shows estimates from multivariable-adjusted without BMI models.

Supplement:

*S3 Table. Associations of global and process-specific polygenic scores with type 2 diabetes risk in secondary analysis*

|  | ***Nurses’ Health Study*** | | ***Health Professionals Follow-up Study*** | | ***Nurses’ Health Study II*** | | ***Combined*** | |
| --- | --- | --- | --- | --- | --- | --- | --- | --- |
|  | ***HR (95%CI)*** | ***P Value*** | ***HR (95%CI)*** | ***P Value*** | ***HR (95%CI)*** | ***P Value*** | ***HR (95%CI)*** | ***P Value*** |
| ***Global polygenic score*** |  |  |  |  |  |  |  |  |
| *Age-adjusted* | *1.36 (1.30, 1.42)* | *<0.001* | *1.34 (1.27, 1.42)* | *<0.001* | *1.69 (1.58, 1.80)* | *<0.001* | *1.42 (1.38, 1.46)* | *<0.001* |
| *Multivariable-adjusted w/ BMI* | *1.28 (1.23, 1.35)* | *<0.001* | *1.27 (1.20, 1.35)* | *<0.001* | *1.51 (1.42, 1.62)* | *<0.001* | *1.33 (1.29, 1.37)* | *<0.001* |
| ***Pathway-specific polygenic scores*** |  |  |  |  |  |  |  |  |
| *Beta-cell dysfunction* |  |  |  |  |  |  |  |  |
| *Age-adjusted* | *1.23 (1.18, 1.28)* | *<0.001* | *1.30 (1.23, 1.38)* | *<0.001* | *1.30 (1.22, 1.39)* | *<0.001* | *1.26 (1.23, 1.30)* | *<0.001* |
| *Multivariable-adjusted w/ BMI* | *1.21 (1.16, 1.27)* | *<0.001* | *1.30 (1.22, 1.37)* | *<0.001* | *1.28 (1.20, 1.37)* | *<0.001* | *1.25 (1.21, 1.29)* | *<0.001* |
| *Impaired insulin synthesis* |  |  |  |  |  |  |  |  |
| *Age-adjusted* | *1.13 (1.08, 1.18)* | *<0.001* | *1.13 (1.07, 1.20)* | *<0.001* | *1.17 (1.10, 1.26)* | *<0.001* | *1.14 (1.11, 1.18)* | *<0.001* |
| *Multivariable-adjusted w/ BMI* | *1.12 (1.08, 1.17)* | *<0.001* | *1.14 (1.07, 1.21)* | *<0.001* | *1.16 (1.09, 1.24)* | *<0.001* | *1.13 (1.10, 1.17)* | *<0.001* |
| *Obesity-mediated insulin resistance* |  |  |  |  |  |  |  |  |
| *Age-adjusted* | *1.07 (1.02, 1.11)* | *0.003* | *1.13 (1.07, 1.20)* | *<0.001* | *1.09 (1.02, 1.17)* | *0.011* | *1.09 (1.06, 1.13)* | *<0.001* |
| *Multivariable-adjusted w/ BMI* | *1.05 (1.01, 1.07)* | *0.021* | *1.13 (1.07, 1.20)* | *<0.001* | *1.07 (1.00, 1.15)* | *0.048* | *1.08 (1.04, 1.11)* | *<0.001* |
| *Body fat distribution* |  |  |  |  |  |  |  |  |
| *Age-adjusted* | *1.24 (1.18, 1.29)* | *<0.001* | *1.24 (1.18, 1.32)* | *<0.001* | *1.28 (1.20, 1.36)* | *<0.001* | *1.25 (1.21, 1.29)* | *<0.001* |
| *Multivariable-adjusted w/ BMI* | *1.21 (1.16, 1.26)* | *<0.001* | *1.25 (1.18, 1.33)* | *<0.001* | *1.23 (1.15, 1.31)* | *<0.001* | *1.22 (1.19, 1.26)* | *<0.001* |
| *Lipid/hepatic metabolism* |  |  |  |  |  |  |  |  |
| *Age-adjusted* | *1.15 (1.07, 1.20)* | *<0.001* | *1.11 (1.03, 1.18)* | *0.004* | *1.11 (1.03, 1.20)* | *0.008* | *1.13 (1.09, 1.17)* | *<0.001* |
| *Multivariable-adjusted w/ BMI* | *1.12 (1.08, 1.17)* | *<0.001* | *1.10 (1.04, 1.17)* | *0.001* | *1.10 (1.03, 1.18)* | *0.004* | *1.11 (1.08, 1.15)* | *<0.001* |

***Table Legend:*** *Hazards ratios and 95% confidence interval of the estimate for type 2 diabetes risk per SD increase in polygenic scores in each of the three prospective cohorts separately and in a combined analysis. Cox proportional hazards models were adjusted for age (in months, continuous) and ancestry-derived principal components (1 to 4) (Age-adjusted model). The multivariable adjusted model without BMI was further adjusted for time-dependent confounders including family history of diabetes (not time-dependent, yes or no), hypertension (yes or no), hypercholesterolemia (yes or no), menopausal status (premenopausal or postmenopausal [never, past, or current menopausal hormone use], women only), smoking status (current, former, never), physical activity (quintiles of MET-hours/week), and total energy intake (quintiles of total caloric intake/day). Fixed-effects inverse-variance weighted meta-analysis was used to combine cohort-specific results.*

Response: Age-adjusted estimates for multivariable-adjsuted models presented before in Fig 2A are now presented in S3 Fig

Supplement:

*S3 Fig: Risk of type 2 diabetes according to categories of the global polygenic scores and adherence to a healthy diet in age-adjusted models.*

***Figure Legend:*** *Shown are adjusted hazard ratios and 95% confidence interval of the estimate for type 2 diabetes according to genetic risk and diet quality categories using the AHEI score. In these comparisons, participants with low genetic risk and high-quality diet served as the reference group. Cox proportional hazards models were adjusted for age (in months, continuous) and ancestry-derived principal components (1 to 4). A fixed-effects meta-analysis was used to combine cohort-specific results.*

Response: The age-adjusted estimates for the association between diet quality and type 2 diabetes risk according to categories of genetic risk is presented in Table 2. This table replaces former Fig 2B and incorporates the secondary analysis without BMI.

Table 2:

***Table 2.*** *Association between diet quality and type 2 diabetes risk according to categories of genetic risk.*

| ***Subgroup*** | ***HR (95%CI)*** | ***P Value*** |
| --- | --- | --- |
| ***Low genetic risk*** |  |  |
| *Age-adjusted model* | *1.74 (1.48, 2.05)* | *<0.001* |
| *Multivariable-adjusted model* | *1.31 (1.11, 1.54)* | *0.001* |
| *Secondary analysis without BMI* | *1.41 (1.17, 1.69)* | *<0.001* |
| ***Intermediate genetic risk*** |  |  |
| *Age-adjusted model* | *1.84 (1.59, 2.13)* | *<0.001* |
| *Multivariable-adjusted model* | *1.39 (1.19, 1.63)* | *<0.001* |
| *Secondary analysis without BMI* | *1.50 (1.29, 1.75)* | *<0.001* |
| ***High genetic risk*** |  |  |
| *Age-adjusted model* | *1.72 (1.53, 1.93)* | *<0.001* |
| *Multivariable-adjusted model* | *1.29 (1.14, 1.46)* | *<0.001* |
| *Secondary analysis without BMI* | *1.38 (1.22, 1.56)* | *<0.001* |

***Table Legend:*** *Hazards ratios and 95% CI for type 2 diabetes risk for low vs high diet quality according to genetic risk categories. Cox proportional hazards models were adjusted for age (in months, continuous) and ancestry-derived principal components (1 to 4) (Age-adjusted model). Multivariable adjusted model was further adjusted for time-dependent confounders including family history of diabetes (not time-dependent, yes or no), hypertension (yes or no), hypercholesterolemia (yes or no), menopausal status (premenopausal or postmenopausal [never, past, or current menopausal hormone use], women only), BMI (quintiles of kg/m^2^), smoking status (current, former, never), physical activity (quintiles of MET-hours/week), and total energy intake (quintiles of total caloric intake/day). A secondary analysis was conducted without adding BMI as a covariate. Fixed-effects inverse-variance weighted meta-analysis was used to combine cohort-specific results.*

**26. Table 2: Please provide the unadjusted analyses, and also please provide both 95% CIs and p values.**

Response: Thank you for this suggestion. We have included the requested information in the supplement.

Supplement:

*S6 Table. Additive interactions between diet quality and genetic susceptibility on type 2 diabetes risk in age-adjusted models*

|  | ***Global polygenic score*** | ***Pathway-specific polygenic scores*** | | | | |
| --- | --- | --- | --- | --- | --- | --- |
|  |  | ***Impaired insulin secretion*** | | ***Impaired insulin sensitivity*** | | |
| ***Polygenic score*** |  | ***Beta, cell dysfunction*** | ***Impaired proinsulin synthesis*** | ***Obesity-mediated insulin resistance*** | ***Body fat distribution*** | ***Lipid/hepatic metabolism*** |
| ***Main effects*** |  |  |  |  |  |  |
| *Diet quality^†^* | *1.50 (1.41, 1.60)* | *1.51 (1.42, 1.61)* | *1.50 (1.41, 1.59)* | *1.49 (1.40, 1.59)* | *1.50 (1.41, 1.60)* | *1.49 (1.40, 1.59)* |
| *Polygenic score^‡^* | *1.43 (1.37, 1.50)* | *1.31 (1.25, 1.37)* | *1.18 (1.12, 1.23)* | *1.08 (1.03, 1.14)* | *1.28 (1.22, 1.34)* | *1.16 (1.10, 1.21)* |
| *Joint effect* | *2.12 (2.01, 2.20)* | *1.88 (1.81, 1.95)* | *1.68 (1.60, 1.75)* | *1.64 (1.57, 1.71)* | *1.86 (1.79, 1.93)* | *1.65 (1.57, 1.72)* |
| ***Relative excess risk due to interaction*** |  |  |  |  |  |  |
| *Relative excess risk due to interaction* | *0.19 (-0.01, 0.38)* | *0.05 (-0.04, 0.15)* | *0.01 (-0.08, 0.10)* | *0.07 (-0.02, 0.15)* | *0.07 (-0.02, 0.17)* | *-0.01 (-0.09, 0.08)* |
| *P value* | *0.061* | *0.257* | *0.834* | *0.120* | *0.122* | *0.891* |
| ***Attributable risk proportion, %*** |  |  |  |  |  |  |
| *Diet quality* | *44.6 (39.7, 49.8)* | *58.5 (52.4, 64.5)* | *73.1 (65.2, 80.9)* | *76.5 (68.2, 84.8)* | *58.4 (52.3, 64.6)* | *76.7 (68.6, 84.8)* |
| *Polygenic score* | *38.8 (33.8, 43.7)* | *35.3 (29.5, 41.1)* | *25.6 (18.2, 32.3)* | *13.1 (4.5, 21.7)* | *32.9 (27, 38.9)* | *24.2 (16.4, 32.1)* |
| *Additive interaction* | *16.5 (-0.5, 34.8)* | *6.2 (-4.2, 16.1)* | *1.4 (-11.4, 14.2)* | *10.3 (-1.9, 22.6)* | *8.6 (-1.7, 18.9)* | *-0.9 (-13.9, 12.1)* |

***Table Legend:*** *Age-adjusted risk of type 2 diabetes estimated from Cox proportional hazards models adjusted for age and ancestry-derived principal components.*

*† Low quality diet vs. high quality diet was defined as a categorical variable based on the median distribution of the diet quality score.*

*‡ Genetic risk defined using the global polygenic score. Estimated effect sizes per SD increase.*

**27. Supplementary Figure 7: Please place the axis labels so it is clear which axis is represented.**

Response: Thank you for this suggestion. We have updated S7 Fig accordingly.

**Comments from the reviewers:**

**Reviewer #1: This manuscript describes a detailed analysis of the potential for an interaction between diet and genetic risk for diabetes in regard to diabetes incidence. The authors conclude that any such interactions must be very small, and that a healthy diet is associated with lower risk of diabetes at all levels of genetic risk.**

**Generally, the results are clearly presented, and the findings are robust. The findings are not surprising for two reasons. First, it is hard to see why high genetic risk would remove the effect of diet. Diet affects blood glucose levels even among people with T1 diabetes, so why would it not do so in people with a high polygenic risk for T2. Second, several other studies (as listed by the authors) have reported the same findings, although the current analysis takes a different and potentially more sophisticated approach.**

Response: We thank Reviewer #1 for the valuable feedback. A major limitation often acknowledged in previos interaction studies is the use of polygenic scores with a limited number of genetic variants. Others argue that aggregating genetic variants affecting different pathophysiological process into a single score is the reason of lack of significant interactions. The latter is particularly relevant in the context of an aetiologically heterogeneous disease as type 2 diabetes. Here we took advantatge of state-of-the-art polygenic scores capturing overall type 2 diabetes risk or distinct pathophysiological processes to ask the question whether genetic risk interacts with diet quality on the risk of type 2 diabetes. In addition, because interactions are scale dependent, and may be absent on one scale (i.e., multiplicative) and present on another (i.e., additive), we systematically evaluated the presence of additive and multiplicative interactions.

Our results concur with previous observations showing that both diet and genetic risk are independently associated with the development of type 2 diabetes without evidence of significant interactions. In the era of precision nutrition, in which genetics and other axes of biological information are used to predict short-term individual postprandrial responses to specific foods or diets, our results are important to emphasize population-wide healthy dietary patterns recommendations for the primary prevention of type 2 diabetes as genetic risk does not seem to modify their effectiveness. We have added the following information:

Page 20, line 370: *Our study adds to knowledge on the interplay between genetic and lifestyle factors by formally investigating whether polygenic scores for type 2 diabetes capturing overall genetic risk or distinct pathophysiological mechanisms could help prioritize individuals who would benefit the most from targeted dietary recommendations.*

Page 20, line 376: *However, previous studies considered a limited number of variants to generate type 2 diabetes polygenic scores, and the lack of significant interactions reported in these studies is often attributed to the mixture of genetic variants affecting different pathways into a single score [6,32]. The latter is particularly relevant in the context of a highly heterogenous disease such as type 2 diabetes, in which groups of individuals might be more likely to develop the disease due to alterations in specific processes.*

Page 21, line 397: *Our findings provide evidence that there is no departure from the additivity of risks attributed to each factor separately, indicating that the presence of the two exposures (low diet and increased genetic risk) does not explain a higher number of cases that could have prevented if only one of the exposures were present.*

Page 21, line 404: *By clarifying that genetic risk and diet quality are each independently associated with the risk of type 2 diabetes and would not have an additive or multiplicative impact on the risk of the disease, our findings can yield useful clinical and public health answers as we prepare for the eventual implementation of precision nutrition.*

Page 21, line 411: *While long-term follow-up studies are needed to better appreciate the value of extremely personalized dietary recommendations for the prevention of diabetes and related metabolic diseases, findings from the present study support public health efforts that emphasize the consumption of healthy dietary patterns.*

**The authors stopped updating dietary information after a diagnosis of CVD or cancer, and carried forward earlier cumulative averages. It is not clear why this reduces bias. If a person develops CVD, and then makes some dietary changes, it may be those exact dietary changes that explain why they didn't subsequently develop diabetes.**

Response: As suggested by the Reviewers and in agreement with this comment, we have now re-ran our analysis without stop updating diet information after the diagnosis of cardiovascular disease or cancer. We edited the methods accordingly.

Page 12, line 225: *To better capture longitudinal trajectories of diet quality, we calculated and used cumulative averages of diet quality. To generate cumulative averages, we continually updated diet throughout duration of follow up.*

**Abstract conclusion. 'gradients of genetic risk' is not quite correct. 'levels of genetic risk' would be better.**

Response: Thank you for this suggestion.

**Table 1. The study legend refers to 'Age standardized characteristics', which may lead the reader to think that they are all standardised to the same age profile. However, the footnote indicates that each analytic sample has been standardised to the age profile of its own parent study. This is confusing. Furthermore, it's not clear what the purpose of this is, and I couldn't see where it was explained in the methods. The purpose of table 1 is to describe the precise population used in the current analysis, not to provide estimates of prevalences and means within the larger study from which the analytic sample has been drawn. Thus, it seems to me that there is no value in age-standardizing.**

Response: Thank you for this suggestion. We have updated Table 1 accordingly and present baseline characteristics without age-standarizing the participant’s characteristics.

**Figure 1. The labelling of the Impaired insulin sensitivity scores is a little confusing. For example, it looks as if score 3 is a genetic risk score for obesity. Also, I would number these 5 pathways 1-2 and 1-3. The current numbering of 1-5 obscures the fact that 3-5 come under the sub-heading of (b).**

Response: Thank you for these suggestions. We have clarified these points in the figure while striving to retain the label used in the original publications to describe these polygenic scores.

**Titles for figures 1 and 2 use different terminology for what appears to be the same concept ('genetic susceptibility' vs 'genetic risk'). Please keep them consistent with each other.**

Response: Thank you for this suggestion. We have updated the titles and used the terms consistently throughout the manuscript.

**Figure 2. It's not easy to tell from the legend how A and B differ from each other. For example, in 2A, the third row compares Low quality diet with High quality diet among people with low genetic risk. This seems to be the same comparison as presented in the first row of 2B. The HRs are the same (1.31), but the CIs and p-values are different.**

Response: Thank you for this suggestion. To clarify our results, in the revised version of the manuscript we placed estimates presented in former figure 2B into Table 2. In addition, we have amended the figure legend accordingly.

**Reviewer #2: The manuscript examines the association of genetic factors and diet quality with the incidence of type 2 diabetes. Novel insight is gained through systematic evaluation of additive and multiplicative interactions between genetic risk and diet quality, which were found to not be significant. Overall, the paper is very well-written and utilizes a number of secondary resources and metrics (e.g., DASH and AHEI scores) to ensure that results are robust and reproducible. The choice of statistical methods is appropriate, and the analysis of each cohort separately ensures that results are not confounded by cross-cohort batch effects. This is a well-executed study, and I only have one relatively minor suggestion.**

Response: We thank Reviewer #2 for the favorable view of our research and for the helpful suggestions to improve the manuscript.

**In the appendix, the authors write that models were adjusted for the first 20 principal components (PCs). However, this raises a concern that if the dominant variance in the data aligns with whether individuals had type 2 diabetes, then such an adjustment would lower the signal-to-noise ratio. Furthermore, the choice of 20 appears to be arbitrary. It would be good to see that a) the top 20 PCs do in fact describe non-random variance in the data (e.g., via Horn's parallel analysis), and b) that the top 20 PCs do NOT have a significant association with type 2 diabetes.**

Response: We used a Bayesian mixed model association method (BOLT-LMM) to run the genome-wide association analysis for type 2 diabetes in UK Biobank (reference). For this analysis, we followed the recommendations from investigators who developed the method and used the the first 20 principal component as covariates to control for false positives while increasing the rate of convergence of the iterative computations performed during the model-fitting steps.

**Minor comments:**

**-The Introduction section is missing its caption**

Response: Thanks, this has been edited.

**-To increase reproducibility and transparency, consider releasing R and SAS code as a publicly available repository (e.g., on GitHub).**

Response: Thank you for this suggestion. Access to statistical code and datasets will be facilitated following the existing data sharing guidelines in NHS, NHS2 and HPFS provided, which can be found on the study websites. We have added the following information:

Page 25, line 477: *Data Availability: Information including the procedures to obtain and access data from the Nurses’ Health Study and Health Professionals Follow-Up Study is described at* [*http://www.nurseshealthstudy.org/researchers*](http://www.nurseshealthstudy.org/researchers) *for the Nurses’ Health Study (contact:* [*nhsaccess@channing.harvard.edu*](mailto:nhsaccess@channing.harvard.edu)*) or* [*https://www.hsph.harvard.edu/hpfs/hpfs_collaborators.htm*](https://www.hsph.harvard.edu/hpfs/hpfs_collaborators.htm) *for the Health Professionals Follow-up Study (contact:* [*hpfs@hsph.harvard.edu*](mailto:hpfs@hsph.harvard.edu)*). Access to statistical codes and datasets will be facilitated following the existing data sharing guidelines provided, which can be found on the study websites.*

**-For completeness, please list the overall correlation between DASH and AHEI scores in each cohort.**

Response: We have included the following information:

Page 17, line 321: *The correlation between the AHEI and the DASH score was high (r^2^ >0.6 in all three cohorts, P <0.001).*

**-In Figure 2 caption, "..participants with low genetic risk and high-quality diet (Fig 2A) or high quality diet (Fig 2B) served as the reference group." seems unnecessarily redundant. Consider simplifying to "..participants with low genetic risk and high-quality diet served as the reference group."**

Response: Thank you for this suggestion, we have updated this figure captation accordingly.

**Reviewer #3: Merino et al examined the joint effects of diet quality and genetic risk on incident T2D in three large prospective cohorts followed up for 902,386 person-years with available genetic data. This is a timely topic given the emerging concept of precision prevention with the implication that individuals with high genetic risk may benefit more from intervention. The authors leveraged the comprehensive genotype and phenotype data in UK Biobank to construct and validate a global polygenic score, as well as several pathway-specific polygenic scores for T2D, which were then analyzed jointly with the diet quality scores quantified by two different methods. The authors also conducted a three-way interaction analysis to investigate whether changes in BMI could modify the joint association of genetic risk and diet quality with T2D. The authors confirmed the independent associations of incident T2D with diet quality and genetic risk albeit without significant interaction and was not modified by BMI. They also quantified that 50% of the proportion of diabetes risk was explained by genetic risk and 38% by high quality diet. Within the polygenic score, ~60% was attributable to beta-cell dysfunction, although sub-analysis showed similar joint associations of quality diet and different pathway-specific polygenic risk scores. Overall, this is a well-conducted study in terms of design, sample size, cohort selection, exposure and outcome definition, and sensitivity analyses although some issues to be addressed.**

Response: We thank Reviewer #3 for the favorable view of our study and for the helpful suggestions to improve the manuscript.

**Major issues:**

**One of the major limitations of this study is the applicability of the results to the general population since the majority of these subjects were well informed and educated healthcare professionals and predominantly nurses. Given the importance of education on diabetes risk, this point had not been discussed. In table 1, the author should state specifically the single sex nature in the 3 cohorts including 35,759 men and women. Although the authors concluded that high quality diet was associated with reduced risk of T2D irrespective of genetic burden, they proposed the use of polygenic score for surveillance purpose but did not discuss the implication of high quality diet within this context.**

Response. Thank you for this suggestion. We concur with Reviewer’s #3 comment that there are limitations to our study that are important to acknowledge and discuss. The study population might not be representative of the US population as participants were mainly well informed and educated healthcare professionals without major chronic diseases at baseline. Thus, it is possible that our results may underestimate the joint association of diet quality and genetic risk with type 2 diabates risk among more vulnerable groups. Our findings need to be confirmed or even expanded in additional studies

Page 23, line 446: *The inclusion of well informed and educated healthcare professionals without major chronic diseases at baseline might limit the generalizability of our findings to other populations.*

***Background***

1) **Page 4, line 71-74: the authors listed four limitations in existing studies that prevented the researchers from identifying “genotypes interacting with lifestyle or dietary exposures” but did not address them in their analysis or discussion.**

- **Limitation 1: *Partial characterization of genetic risk due to a limited number of variants*. There are other genetic loci associated with other traits that may interact with diet or lifestyle on T2D genetic risk (e.g.** [**https://pubmed.ncbi.nlm.nih.gov/33864366/**](https://pubmed.ncbi.nlm.nih.gov/33864366/) **reported gene-diet interactions that influenced HbA1c ). The “partial characterization of genetic risk” is not only restricted to “limited number of variants” but rather the specificity, sensitivity and effect size of these scores. In Figure 1, the “Beta-cell dysfunction” score constructed from fewer of SNPs compared with the “850,000 independent variants” used for the global polygenic score, yielded comparable hazard ratios on incident T2D. Thus, increasing the number of T2D risk loci does not necessarily provide additional variance in discriminating subjects with different T2D genetic risk. In the Appendix, the global polygenic score for T2D together with sex and age had an AUC of 0.638, what is the AUC when they are combined with the dietary scores in explaining the total variance? Given the differences in genetic architecture and many non-genetic factors across various populations and settings, the continuing focus of using aggregated polygenic risk score to detect gene-environment interaction continues to impede our understanding in the effects of environment-personal-lifestyle-familial factors on the development of T2D.**
- **Limitation 2: *Interactions in previous studies were predominantly assessed on the multiplicative scale alone*. The authors only presented the additive Relative Excess Risk due to Interaction (RERI) in Table 2. The authors should include the index estimates with confidence intervals and *P* values of multiplicative interactions for completeness.**
- **Limitation 3: *Single time point dietary exposure*. The cohorts had multiple diet quality scores collected at different time points. The authors should state clearly i) whether the diet quality scores were used as a time-fixed variable or a time-varying variable in the Cox model; ii) compared with the single time point diet quality score, e.g. the last one before endpoint, what was the advantage of the cumulative average? Was the cumulative average sufficient to capture the longitudinal trajectory of the score?**
- **Limitation 4: *Limited follow-up*. The three cohorts were followed up from 26 years to 30 years. With such long follow-up, considerable changes in the socioeconomic and demographic characteristics and ecological factors could influence greatly on diet quality and T2D incidence. Did the author adjust for any socioeconomic covariates in their Cox regression analyses if there were relevant information in the questionnaire, or discuss the issue if not available. Did they adjust for year of data capture?**

Response: Thank you for bringing these aspects.

Regarding the the partial characterization of genetic risk, it is important to emphasize that when conducting this type of interaction studies, a crucial decision is whether to restrict genetic information to single variants or aggregate different signals into a polygenic score that capture the overall genetic risk. Both approaches can be easily justified based on the specific research question. The rationale to use a global polygenic score in this study relies on the observation that, similar to other metabolic diseases such as obesity or coronary artery disease, for the majority of individuals with type 2 diabetes, genetic susceptibility results from the cumulative effects of numerous variants with modest effects. In addition, given the aetiological heterogeneity that characterizes type 2 diabetes, we used recently developed pathway-specific polygenic scores with variants that share similar pathophysiological characterisitcs. Our approach clearly expands beyond previous interaction studies and provides convinding data on the interplay between genetics and diet in type 2 diabetes. In response to the comments above, we have re-written the discussion and limitations. In addition, we have added the following information:

Page 22, line 431: *We computed global and pathway-specific polygenic scores for type 2 diabetes, but the use of aggregated scores might have missed potential interactions driven by highly penetrant single genetic variants of strong effects or variants for glycemic traits interacting with environmental exposures. However, further restraining the number of genetic variants will limit the clinical and public health value of our findings as highly penetrant variants tend to be rare or extremely rare in the general population*.

Estimated effect sizes for multiplicative interactions were initially provided in S7 Table. Following Reviewer’s #3 suggestion but we have placed them in Table 3.

The diet quality score was used as a time-varying exposure starting at 1986 in NHS and HPFS and 1991 in NHSII and every 4 years thereafter. A previous study comparing cumulative averages vs a single time point estimate suggest that the single measurement of dietary data in the baseline analysis leads to attenuation of the association and that this measure is more prone to measurement error than the cumulative average (Bernstein et al. 2011). The cumulative average instead of a single time point (i.e. baseline) could be considered a proxy of long-term diet intake and it can also minimize within-person variation and it is a common approach for longitudinal analysis with repeated measurements in dietary analysis. We have added the following information:

Page 12, line 224: *To better capture longitudinal trajectories of diet quality, we calculated and used cumulative averages of diet quality. To generate cumulative averages, we continually updated diet throughout duration of follow up.*

Specific information on socioeconomic status or ecological factors is not available throughout the follow-up. Unfortunaltey, we could not account for changes in socioeconomic status in our models.

***Methods***

2) **For the global polygenic risk scores and pathway-specific risk scores, suggest**

- **present the distribution of these PRS in the three cohorts separately;**
- **show overlap between the SNPs in the global and pathway-specific PRS;**
- **show correlation among the global PRS and pathway-specific PRSs**
- **discuss AUC differences between UK Biobank validation dataset (0.723) and combined cohort under study (0.638)**

Response: Thanks for these suggestions. We have included the distribution of the polygenic scores in S1 Fig.

Page 16, line 296: *The polygenic scores were normally distributed (S1 Fig).*

All genetic variants included in the pathway-specific polygenic scores were also present in the global polygenic score. A key aspect from our study is that weights to generate these two sets of polygenic scores came from very different approaches. While weights to generate the global polygenic score were based on their association with type 2 diabetes in the GWAS conducted among participants in UK Biobank, genetic variants included in the pathway-specific polygenic scores were weighted by the prior to belong to a given pathophysiological process. In other words, the weights of given genetic variant that was simulatenously included in the global and pathway specific polygenic scores are different as they represent different aspects of genetic risk. As a results, the correlation between polygenic scores included in this study is low. Genetic variants and weights to generate the pathway specific polygenic scores were initially provided in S1 Table, and more details about the approach to generate these scores can be found in the original publication (Udler MS. *et al.,* PLoS Medicine 2018). We have the following information:

Page 17, line 309: *The correlations between polygenic scores included in this study were modest (r^2^ ranging from 0.27 to 0.07), supporting the notion that they capture different axes of genetic predisposition (S4 Table).*

Finally, the AUC in UK Biobank is likely inflated due to shared non-genetic factors and random noise as this analysis was conducted in an internal validation set.

**Regarding the diet quality assessed at baseline and every 4 years thereafter,**

- **What was the average number of diet quality scores for a subject in the three cohorts respectively?**
- **Was there any correlation between the number of diet quality scores and the cumulative average of the scores (i.e. whether the subjects with more diet quality scores available tended to have a higher cumulative average)? If so, this may be a marker of adherence and would that affect the interaction?**
- **What was the within-subject variance distribution in the three cohorts respectively? Is the cumulative average a valid surrogate of the long-term diet trajectory for subjects especially in those with fluctuating diet quality and large within-subject variance.**
- **Was there any difference in the within-subject variance among the three cohorts?**
- **The authors stopped updating dietary information when the subjects first reported having cardiovascular events or cancers. What about other factors that could dramatically change subjects’ diet habits (e.g. T2D education or elevated glycemic traits that the subject may be aware of)?**
- **Did family history (FH) of diabetes affect the diet quality scores? Genetics is only a component of FH, did the authors repeat the analysis in those with or without FH?**

Response: In NHS and HPFS the total number of available food frequency questionnaires (FFQ) was up to 7 (from 1986 to 2010 every four years). The mean (SD) of FFQ count in NHS was 4 (2) and in HPFS 3 (2). In NHS II the total number of available FFQ was up to 6 (1991 to 2011 every 4 years) and the mean (SD) of FFQ count was 3 (2). The correlation between the number of diet scores and the cumulative average score was low in all cohorts. In NHS the correlation was 0.14, in NHS2 was 0.20, and in HPFS was 0.11, indicating that the number of FFQ is independent of adherence to healthy diet quality and unlikely to affect the interaction. Also, no substantial within-subject variance among subjects was noted. As mentioned above, the use of cumulative average instead of a single time point (i.e. baseline) could be considered a proxy of long-term diet intake and it can also minimize within-person variation and it is a common approach for longitudinal analysis with repeated measurements. As suggested by Reviewer #1, we have not stop updating dietary information in the revised version of the manuscript. However, results are consistent with previous stop updating dietary information results. We did not find differences in the diet quality score based on family history of diabetes (i.e., in NHS the mean (SD) diet quality score was 53.8 (9.9) among people with family history of diabetes and 53.8 (9.9) among those without). Unfortunately, T2D education or elevated glycemic traits are not available in this dataset.

3) **Page 6, line 183: The statement “Models were adjusted for time-varying …” is very vague. Please define the time-varying covariates and the “a priori” rationale for considering them as time-varying risk factors. Similarly, what were the time-fixed covariates in the models and the values at which time-point were used. A conceptual framework including these mediators or confounders would be helpful in explaining the selection of these covariates in the model.**

Response: We thank the Reviewer for this point. This has now been clarified in the methods section.

Page 13, line 236: *We have used as time-varying variables in the models, the variables that change over time including history of hypertension, history of hypercholesterolemia, menopausal status, BMI, smoking status, physical activity (quintiles of MET-hours/week), and total energy intake. Family history of type 2 diabetes and PCs are considered time-fixed variables at baseline. For the variables that were updated throughout the follow-up the questionnaire year determined the time point.*

4) **Page 6, line 185: The statement “The fully adjusted model was further adjusted …” is contradictory.**

Response: Thanks for this suggestion, this has been edited.

Page 13, line 243: *The multivariable adjusted model was further adjusted for […].*

5) **Page 6, line 190-191: “Because time varying BMI could be on the causal pathway between diet quality and …, we also conducted separate models without adjusting for BMI”. Where were the results of these separate models? In supplementary figure 7, the results are presented in 3 categories of BMI, was this the baseline BMI rater than changes in BMI between baseline and censor point?**

Response: Thanks for this suggestion. The results without adjusting the models for BMI were consistent with the primary models (please see Table 3, and S3 Table).

6) **Page 10, line 200: What was the “median distribution of the diet quality score in each cohort”? The statement is confusing from a statistical aspect.**

Response: We edited this statement

Page 14, line 265: *based on the median of the diet quality score in each cohort.*

7) **Page 10, line 200: The RERI could also be calculated on a multiplicative scale to provide the index estimates of multiplicative interaction.**

Response: Thank for this suggestion.

8) **Page 10, line 205: “tested for multiplicative interactions by comparing the -2 log likelihood”. The statement “-2 log likelihood” was informal. In addition, what test was used?**

Response: Thanks for this suggestion. To test for multiplicative inteactions we used the log-likelihood ratio test to compare the goodness of fit of a multivariable adjusted model with and without the cross-product interaction term.

Page 14, line 262: *We tested for multiplicative interactions using the log-likelihood ratio test to compare the goodness of fit of a multivariable adjusted model with and without the cross-product interaction term [27].*

9) **Page 10, line 209: The statement “examine whether changes in BMI modified the joint association …” is also vague. Which one was used to perform the 3-way interaction, time varying BMI or changes between two BMI?**

Response: We used time varying BMI for these analyses. We have added the following information:

Page 15, line 274: *Second, we conducted 3-way interaction analyses to examine whether BMI modified the joint association between increased genetic risk and low diet quality on type 2 diabetes risk.*

***Results***

10) **In Table 1, please include the incidence of T2D, incidence of death, number of lost to follow up and number of cardiovascular/cancer events in the three cohorts.**

Response: A total of 4,433 participants developed type 2 diabetes during follow-up (n=2,204 (15.2%) in NHS, n=1,285 (13.6%) in HPFS, and n=944 (7.9%) in NHS II). The number of cardiovascular events was 6,105 (n=3,481 (25.1%) in NHS, n=2,437 (25.8%) in HPFS, and n=1,187 (9.9%) in NHS II). The number of incident cancers was 4,524 (n=1,980 (1.7%) in NHS, n=1,130 (12%) in HPFS, and n=1,414 (11.9%) in NHS II). The number of deaths was 673 in all three cohorts. The low mortality rate among included participants can be explained by the fact that participants had to be alive in 2011 when genetic determinations were performed. As stated in the manuscript, missing data was below than 5%. We have included the following information:

Page 16, line 293: *A total of 4,433 participants developed type 2 diabetes during 902,387 person-years of follow-up (n=2,204 (15.2%) in NHS, n=1,285 (13.6%) in HPFS, and n=944 (7.9%) in NHS II).*

11) **Page 12, line 246: “there was a risk gradient with increasing …”. Please perform trend analysis to demonstrate the statistical significance of such “risk gradient”.**

Response: Thank you for this suggestion, the estimates showing the risk gradient and their respective p-values were initially included in the figure.

12) **Page 12, line 251-252: “The available sample gave us 80% statistical power …”, at what significance level?**

Response: We have clarified this.

Page 14, line 260: *The available sample gave us 80% statistical power at α 0.05 […]*

13) **Given that the three cohorts were all sex-specific (two women cohorts and one men cohort), did the authors observe any sex-specific association between the risk factors under study and T2D incidence?**

Response: We did not investigate potential sex-specific association of diet quality and genetic risk with T2D incidence.

14) **The lack of interactions between T2D genetic risk scores and diet quality on incident T2D limited the novelty of this work. A recent study reported interaction between fruit intake and T2D genetic risk (**[**https://pubmed.ncbi.nlm.nih.gov/33399975/**](https://pubmed.ncbi.nlm.nih.gov/33399975/)**). Did the author explore the association of a subset of foods or nutrients well known for their risk-conferring or risk-reducing effect for T2D instead of using the aggregated score .**

Response: Thank you for this suggestion. We did not consider investigating the interaction of specific foods or nutrients with genetic risk on the development of type 2 diabetes as dietary patterns better capture the complexity of food intakes than any one individual food or nutrient, and offer the advantage of describing usual consumption of foods in typical diets.

The study highlighted by Reviewer #3 is a cross-sectional study. The study design precludes the main conclusion that the genetic association of T2D could be alleviated by increased fruit intake. In addition several methodological limitations exists in this study, including the use of a very limited number of genetic variants to characterize type 2 diabetes genetic risk. A GWAS study published in 2020 identified 183 loci associated with T2D in East Asian Individuals (Spracklen CN, Nature 2020), but in the suggested study polygenic scores were built based on information of 34 loci.

15) **Have the authors performed GWAS on dietary traits or borrowed similar information from the UK Biobank analysis (**[**https://pubmed.ncbi.nlm.nih.gov/32193382/**](https://pubmed.ncbi.nlm.nih.gov/32193382/)**) to investigate whether there are shared genetic factors between T2D and unhealthy diet. Other similar work such as genome-wide gene-diet interaction analysis should preferably be performed or cited to reflect the limitation of the study (**[**https://pubmed.ncbi.nlm.nih.gov/33864366/**](https://pubmed.ncbi.nlm.nih.gov/33864366/)**). These analyses would complement the existing results and strengthen this study.**

Reponse: No, we did not run a GWAS for dietary traits in our study. The study by Westerman KE, *et al.,* Hum Mol Genet 2021 identified a handful of genetic variants interacting with food components on glycemic phenotypes. However, the study used cross-sectional data, which is prone to residual confounding and reverse causation. In addition, given the extreme polygenicity of glycemic phenotypes with hundreds of genetic loci modestly influencing A1c, the clinical value and relevance of such information is likely to be modest.

16) **Limitations and strengths of the study should be discussed.**

Response: Thank you for this suggestion. We have added the following information:

Page 22, line 415: *The strengths of this study include the use of new generation polygenic scores for type 2 diabetes that capture overall genetic risk or specific pathophysiological processes, the well validated measures of dietary factors and the use of repeated diet measurements to reduce measurement error and noise, the large number of incident type 2 diabetes cases and extended follow-up, and the consistency of our findings in sensitivity analyses. This is the first large long-term interaction study using both global and pathway specific polygenic scores to systematically investigate the presence of additive and multiplicative interactions between genetic risk and diet quality on the development of type 2 diabetes.*

*We acknowledge several limitations. Because this was an observational study and allocation to low- or high-quality diet was not randomized, we could not infer causality regarding the associations of low diet quality and increased genetic risk on the development of type 2 diabetes. A possible reason for the lack of interaction between the polygenic risk score and diet on the risk of type 2 diabetes could be imprecision in dietary intake measurement. We used cumulative averages of diet, which yield more precise dietary intake estimates than baseline intakes alone[40] but the use of more objective dietary intake assessment methods such as the use of smartphone applications, wearable technology, or dietary intake biomarkers, is necessary to accurately ascertain dietary intake and reduce self-reported errors [39,41]. We computed global and pathway-specific polygenic scores for type 2 diabetes, but the use of aggregated scores might have missed potential interactions driven by highly penetrant single genetic variants of strong effects or variants for glycemic traits interacting with environmental exposures [42]. However, further restraining the number of genetic variants will limit the clinical and public health value of our findings as highly penetrant variants tend to be rare or extremely rare in the population. In addition, the use of tails of polygenic score distribution (i.e., top 5% or 1% of genetic risk) could be used to detect potential interactions more likely to be present among people with very high or low genetic risk. However, such analysis is likely to yield spurious interactions due to unbalanced covariates and residual confounding, and it might be unpowered compared to the assessment of interaction in the continuous scale [43]. We restricted our analyses to participants for whom genetic data were available, which represents a small proportion of the original sample and might have induced selection bias. Participants for genetic determinations were selected to be representative of the original study population, and demographic characteristics and health status of participants with genetic information were generally similar to those who did not. The inclusion of well informed and educated healthcare professionals without major chronic diseases at baseline might limit the generalizability of our findings to other populations. However, increased genetic risk and low diet quality have been associated with risk of type 2 diabetes in other populations [11].*

**Minor issues:**

1) **Page 4, line 82: “processes such impaired insulin …” 🡺 “processes such as impaired insulin …”**
2) **Page 6, line 116: “… a global polygenic score for type 2 diabetes that capture overall …” 🡺 “that captures overall …”**
3) **Page 7, line 138: “transcriptional regulation (13)’ indicating …” 🡺 “transcriptional regulation (13), indicating …”**
4) **Page 7, line 144-145: “in this study with different the number of …” 🡺 “in this study with different number of”**
5) **Page 7, line 154: “we used the DASH score (Dietary Approaches to Stop Hypertension) …” 🡺 “we used the Dietary Approaches to Stop Hypertension (DASH) score …”**
6) **Page 10, line 202: “which is the proportion of risk duet to …” 🡺 “… due to …”**
7) **Page 26, line 566: “… or median (IQR) …” 🡺 “… or medians (IQR) …”**
8) **Page 27, line 577: “time varying confounders including age, ancestry-derived principal components, family history …”. How could the genetic principal components be used as time varying covariates? As for family history, can change in status over time influence dietary habit and thence risk of diabetes.**

Response: We thank the Reviewer for these suggestions. We have amended all these minor issues and clarified that ancestry derived principal components and family history of diabetes were not time-varying.

Page 13, line 236: *We have used as time-varying variables in the models, the variables that change over time including history of hypertension, history of hypercholesterolemia, menopausal status, BMI, smoking status, physical activity, and total energy intake. Family history of type 2 diabetes and ancestry-derived principal components were considered time-fixed variables at baseline.*

**Reviewer #4: The paper describes a prospective cohort study of interaction between genetic risk and diet quality on risk of type 2 diabetes. Three studies that followed up health professional's for over 900,000 person years were studied. Association is found with polygenic risk scores and with diet quality. These appear to additively affect risk of Type 2 diabetes with no evidence of interaction on the additive or multiplicative scale.**

**This is a well done study and the work is well described. It provides further support for the idea that lifestyle modification is important independent of genetic risk.**

Response: We thank Reviewer #4 for the favorable view of our research and for the helpful suggestions to improve the manuscript.

**1. While I think the paper has useful findings, one concern I have is how much of an advance this is over the previous papers (referenced here from 5 to 11). It seems this lack of interaction between genetics and lifestyle has been well described several times in other studies, which is acknowledged in the paper. I think the main new finding claimed in this paper is the lack of additive interaction - but this seems like a limited advance over the previous studies. I would like more discussion - for a non-statistician - on the reasons why this is an important finding.**

Response: Thank you for this relevant suggestion. While previous studies have already described the lack of interaction between genetic and lifestyle factors on the risk of type 2 diabetes, a major limitation acknowledged in these studies is the partial characterization of genetic risk due to the continuos discovery of new genomic regions associated with type 2 diabetes. Others argue that the combination of genetic variants into a single score without any information about their physiological relevance is the main reason for lack of interactions reported in these study. This is particularly relevant in the context of a highly heterogeneous disease such as diabetes.

Our study adds to knowledge by formally investigating whether polygenic scores for type 2 diabetes capturing overall genetic risk or distinct pathophysiological mechanisms could help prioritize individuals who would benefit the most from targeted dietary recommendations. We have highlighted now in the discussion that our results showing no evidence of additive or multiplicative interactions between diet and genetic risk on the development of type 2 diabetes is crucial to inform more efficient public health strategies.

As we enter in the era of precision medicine, we believe these results are important to support public health efforts that emphasize population-wide healthy dietary patterns for the primary prevention of type 2 diabetes as genetic risk does not seem to modify their effectiveness. Further, our results emphasize the value of genetic risk assessment to identify individuals at increased disease risk and their potential for risk stratification and surveillance. This information is now included in the new discussion.

**2. I also wonder why the UK Biobank study wasn't used in this study for comparison. That is also a longitudinal study and is used here to generate genetic risk scores, but I'm unsure why it also couldn't have been used for the main analysis - it would at least provide a comparison to a different population with different study selection criteria.**

Response: Thank you for this suggestion. While we agree that adding data from UK Biobank in our study would serve as a comparision of our findings to a different study population, the generation of the diet quality score is challenging in UK Biobank as essential items to generate the score are not available and not enough detailed dietary information is available. The table below describes which components of the AHEI score are available or not available in the UK Biobank.

**3. Diet quality is, by its nature, not a precise measure. The manuscript describes a good approach to assessing diet quality, but if you measure something imperfectly then this measurement error is going to make the possibility of finding interactions much harder. Do the authors think that this is a potential explanation for the lack of interactions identified?**

Response: We agree with Reviewer #4 that measurement error might be one of the reasons we were not able to identify interactions between diet and genetic risk on the development of type 2 diabetes. To partially mitigate this, as some error and noise is inevitable, we used approaches to reduce self-reported dietary intake bias such as the use of repeated measures of diet to reduce error and account for within person variability. In addition we excluded participants over-reporting or under-reporting diet, and used standard epidemiologic modelling adjusting for potential confounders including energy, age, sex, body size, physical activity. As acknowledged in the limitations, the use of more objective dietary intake assessment methods such as the use of smartphone applications, wearable technology, or dietary intake biomarkers, is necessary to accurately ascertain dietary intake and reduce self-reported errors. We have added the following information:

Page 22, line 426: *A possible reason for the lack of interaction between the polygenic risk score and diet on the risk of type 2 diabetes could be imprecision in dietary intake measurement. We used cumulative averages of diet, which yield more precise dietary intake estimates than baseline intakes alone[40] but the use of more objective dietary intake assessment methods such as the use of smartphone applications, wearable technology, or dietary intake biomarkers, is necessary to accurately ascertain dietary intake and reduce self-reported errors [39,41].*

**4. The extreme of polygenic risk aren't assessed here (e.g.>5% or >1% tails of polygenic risk). While overall in the population there may be no obvious interactions, what about if someone has a very high genetic risk of diabetes? In these individuals you might imagine diet does have a less of an impact? Has this been tested?**

Response: While the study of people at extreme genetic risk is a great suggestion, and something that has been used in the field of polygenic socres recently, there are some methodological concerns on these type of analyses. First, the use of the top 5 or 10% tail of polygenic score distribution would likely reduce the statistical power to detect interactions. If true interactions exist, we should have detected them in the continuos scale as statistical power is higher than in subgroup analysis. Second, by generating random groups of polygenic score distribution, there is the possibility to add noise or confounding due to unbalanced covariates. We have added the following information:

Page 23, line 437: *In addition, the use of tails of polygenic score distribution (i.e., top 5% or 1% of genetic risk) could be used to detect potential interactions more likely to be present among people with very high or low genetic risk. However, such analysis would have lower statistical power compared to the assessment of interaction in the continuous scale, and it might yield spurious interactions due to unbalanced covariates between groups and residual confounding [43].*
