# Supplementary material for: Polygenic scores, diet quality, and type 2 diabetes risk: An observational study among 35,759 adults from 3 US cohorts
Source: PLoS Med. 2022 Apr 26;19(4):e1003972. doi: 10.1371/journal.pmed.1003972 (PMC9041832; doi:10.1371/journal.pmed.1003972)
Supplement: Attachment — Submitted filename: Response.PMEDICINE-D-21-01465R2.FINAL.docx [file pmed.1003972.s027.docx]

**EDITOR COMMENTS:**

**1. Please address the remaining points of reviewer 2, including the issue of including top principal components in the model, as well as acknowledging grant support and providing source code and all underlying data needed for supporting these analyses.**

Response: Thank you for the valuable feedback. We have addressed the remaining points.

**2. Title:  We suggest removing the word prospective from the title. We suggest including the study setting/population in the title.**

Response: Thank you for this segguestion. We have amended the title accordingly

Page 1, line 1: *Polygenic scores, diet quality, and type 2 diabetes risk: an observational study among 35,759 adults from three US cohorts.*

**3. Data availability statement: Please revise the data statement, and please update this in the Data Availabilty section of the manuscript submission system. PLOS Medicine requires that the de-identified data underlying the specific results in a published article be made available, without restrictions on access, in a public repository or as Supporting Information at the time of article publication, provided it is legal and ethical to do so. Please describe the locations of code and datasets necessary for replication of the analyses, or contact information for how these may be obtained. If the statistical codes and datasets are not freely available, please describe briefly the ethical, legal, or contractual restriction that prevents you from sharing those. Please also include an appropriate contact (web or email address) for inquiries (please note, this cannot be a study author).**

Response: Thank you for guiding us on this. We share PLOS Medicine’s principle that sharing data and code is necessary to solve the replication crisis we face in science and foster scientific progress. We have added the appropriate information in the manuscript submission system.

**4. Abstract: Background: At line 36, we suggest removing the word “prospectively” from the sentence.**

Response: Thank you for this suggestion, this word has been removed.

**5. Methods: Lines 128-131: Please provide either data or a reference supporting these sentences: “Participants for genetic determinations were selected to represent a representative sample of the original sample. Demographic characteristics and health status of participants with genetic information were generally similar to those who did not, therefore bias due to selection are probably minimized.”**

Response: We have added the following information and amended these sentences.

Page 8, line 129: *Participants for genetic determinations were selected to represent a representative sample of the original sample. Demographic characteristics and health status of participants with genetic information were generally similar to those without genetic information (S1 Table).*

**6. Discussion: Line 419: Please temper this statement with “To the best of our knowledge, this is the first long-term…”**

Response: Thank you for this suggestion. We have added the following information.

Page 22, line 420: *Further, we generated both global and pathway specific polygenic scores to systematically investigate the presence of additive and multiplicative interactions between genetic risk and diet quality on the development of type 2 diabetes.*

**7. Page 25: Please remove the Author Contributions, Prior Presentation, and Data Availability sections from the main text, and please be sure all relevant information is entered in the manuscript submission system.**

Response: Thank you again for guiding us on this and please accept our most sincere apologies for any formatting issues.

**8. References: Please check the formatting of each reference, including journal title abbreviations. For example, reference 6 should be “PLoS Med” and reference 7 should be “Nat Commun”. Please use the "Vancouver" style for reference formatting,**

Response: We confirm we have checked the formatting of each reference and that used Vancouver style for reference formatting.

**9. Figure 1 and Figure 2: Please also provide the unadjusted results. Please define all abbreviations in the legend (e.g. NHS, HPFS).**

Response: While we appreciate the suggestion to provide unadjusted estimates, genetic epidemiology studies requiere the use of minimally adjusted covariates to avoid potential bias such as population stratification. Population stratification occurs when there are systematic ancestry differences in allele frequencies between case subjects and controls subjects. Our crude model is now stratified by age and adjusted for ancestry derived principal components. We confirm that all abbreviations displayed in Fig 1 and Fig 2 are defined in legend.

**10. STROBE Checklist: Thank you for including the checklist. Please note “Funding Statement” as the location for item 22 “Funding” on the checklist.**

Response: Thank you for your advice on this.

**11. S1 Appendix: Type 2 diabetes polygenic scores: Please define NHS I, NHS II and HPFS at first use in the text. Please also clarify “Genetic variants included in each polygenic score and corresponding weights for each variant are detailed in the appendix (pp).” Please also format references using “Vancouver” style, as in the main text.**

Response: Thank you for these suggestions. We have defined NHS, NHS II and HPF in S1 appendix, clarified this sentence (“*Genetic variants to compute these polygenic scores and their respective weights are detailed in S2 Table.”*) and formatted references using Vancouver style.

**12. S1 Table: In the legend, please define all abbreviations in the table.**

Response: Thank you for this suggestion. We have now defined all abbreviations in the new S2 Table.

**13. S4 Table: Please define NHS I, NHS II and HPFS in the legend.**

Response: Thank you. We have now defined these abbreviations in the legend.

**14. S9 Figure: Please define all abbreviations used in the figure in the legend.**

Response: We confirm all abreviations used in S9 Fig are defined in the legend.

**Comments from the reviewers:**

**Reviewer #1: Thank you for addressing my comments.**

Response: We thank Reviewer #1 for helping us improve the manuscript.

**Reviewer #2: Thank you for the opportunity to review the revised version of this work. Unfortunately, I feel that the authors have largely ignored my concerns in favor of working to address the more extensive criticism provided by the other reviewers.**

**In the response, the authors write that they followed the recommendations from investigators who developed BOLT-LMM. However, these recommendations are not quoted entirely accurately. Including principal components (PCs) as covariates to control for false positives is generally recommended by the BOLT-LMM developers for linear regression models, based on their earlier work [PMID: 16862161]. For mixed-model analysis, such as the one employed here, the original work describing BOLT-LMM states that "principal component analysis is not part of BOLT-LMM; it is unnecessary to perform PCA when running mixed model association methods" [PMID: 25642633]. Their later work does suggest "including PC covariates for the purpose of accelerating convergence of [...] BOLT-LMM" [PMID: 29892013], but algorithm runtime is of low importance in a one-shot analysis, especially when the source code is not being released for future reproducibility (see below). Blindly following a recommendation to improve runtime does nothing to address the original concern that the top principal components may have real (i.e., not ancestry-confounded false positive) type 2 diabetes signal, and that adjusting for these PCs may unnecessarily lower signal-to-noise ratio.**

Response: We appreciate Reviewer’s #2 comment and would applogy if it appeared that the previous comments were not fully addressed.

We understood that the key concern of this comment was whether adjusting for top genetic principal components (PCs) would reduce statistical power of genetic associations in GWAS. As Reviewer #2 pointed out, adjusting for top PCs has been a routine approach in GWAS to account for population stratification [PMID: 16862161]. Due to the small effect size of individual genetic variants on complex phenotypes, and that PCs are defined by many unliked genetic markers, genetic studies have been consistently adjust for top PCS as this approach is unlikely to explain away true genetic associations (please see the most recent DIAGRAM consortium GWAS for T2D, Mahajan 2018 Nature Genetics PMID: 30297969, adjusting top 6 PCs in UK Biobank). Adjusting for top PCs in BOLT-LMM might seem unnecessary for the mixed model, but it did not hurt the statistical power, The updated version of BOLT-LMM [PMID: 29892013] safely recommend adjusting for top PCs to greatly improve run-time without compromising statistical power. The Suppl. Fig. 2 in this same paper showed that BOL-LMM without adjusting for PCs was less calibrated than linear regression adjusting for PCs, though the authors stated that the difference was acceptable. Mathieson, McVean 2012 Nature Genetics (PMID: 22306651) also showed that adjusting for more PCs could potentially remove association bias due to patterns of stratification by low frequent variants that could not be accounted for by mixed model approaches. Further, because top PCs represent population ancestry (strongly related to dietary habits), it is particularly important to account for them before any downstream gene-diet interaction analysis. For example, Abdullah Said 2018 JAMA Cardiology (PMID: 29955826) adjusted for top 30 PCs in UK Biobank when examining the effect of genetic and lifestyle interactions on diabetes among UK Biobank participants. Taken together, we consider it is still acceptable to account for top PCs in BOLT-LMM analysis.

**The URLs provided by the authors do NOT address my request to improve transparency and reproducibility of the study. The first URL is broken. The page at the second URL states that "Researchers using the NHS and NHSII data are required to acknowledge the grant support received by the National Institutes of Health (NIH) listed below, as appropriate, in all publications", yet the manuscript itself (which makes extensive use of these data) does not list any grants in the Acknowledgments section. Lastly, holding back source code under the guise of data availability is not appropriate. Data and software are two separate entities, and software licensing follows its own structure that is completely independent of any data usage agreements (**[**https://secure-web.cisco.com/1rdm-oPIjrOkk-ZtUUg5zKBNkfcsBIZlrYjeSIDBL0dkGvAGj7vGYWPcHc0JZisBo9Q0ZGcSWhgP94o3qdlRRZiBWwpLfS2ifugu2IspJOHr9PaNmTnWKK3wCmpZqvy8Oa-5JrxMn2Iyn1HxVoPTSlWIwzEyeACyzLnbDF8G5iXPrkfiTjZAKq0QBm_zHupaxzJImQwFhe58m8Hjh3ebtFXJWYrUA2qO3RnmWXG7gQf5IiFm-uriQceg1og1Og8kRoV39J8Ppdg3wafqL8dgsZQSAJybzyMY_j9mT1WVw6SlLSFzKowSChrOiVsyU5SDK/https%3A%2F%2Fen.wikipedia.org%2Fwiki%2FSoftware_license**](https://secure-web.cisco.com/1rdm-oPIjrOkk-ZtUUg5zKBNkfcsBIZlrYjeSIDBL0dkGvAGj7vGYWPcHc0JZisBo9Q0ZGcSWhgP94o3qdlRRZiBWwpLfS2ifugu2IspJOHr9PaNmTnWKK3wCmpZqvy8Oa-5JrxMn2Iyn1HxVoPTSlWIwzEyeACyzLnbDF8G5iXPrkfiTjZAKq0QBm_zHupaxzJImQwFhe58m8Hjh3ebtFXJWYrUA2qO3RnmWXG7gQf5IiFm-uriQceg1og1Og8kRoV39J8Ppdg3wafqL8dgsZQSAJybzyMY_j9mT1WVw6SlLSFzKowSChrOiVsyU5SDK/https%3A%2F%2Fen.wikipedia.org%2Fwiki%2FSoftware_license)**The authors need to decide what software license applies to their source code. If it falls under the umbrella of open source, then the code should be released as such. If the authors prefer to use a proprietary license, then it may be good to include a brief explanation why sacrificing transparency and reproducibility is appropriate in an NIH-funded study.**

Response: Thank you for this suggestion. Following Associate Editor’s advice, we have added the appropriate information in the manuscript submission system.

**Reviewer #3: The authors have addressed all comments satisfactorily and the paper is much improved**

Response: We thank Reviewer #3 for the valuable feedback.

**Reviewer #4: The authors have addressed my comments well.**

Response: We thank Reviewer #4 for the helpful suggestions to improve the manuscript.
