# Supplementary material for: Polygenic scores, diet quality, and type 2 diabetes risk: An observational study among 35,759 adults from 3 US cohorts
Source: PLoS Med. 2022 Apr 26;19(4):e1003972. doi: 10.1371/journal.pmed.1003972 (PMC9041832; doi:10.1371/journal.pmed.1003972)
Supplement: Attachment — Submitted filename: Response.PMEDICINE-D-21-01465.R3.FINAL.docx [file pmed.1003972.s028.docx]

**EDITOR COMMENTS:**

**1. Reviewer 2 comments: As suggested, please complete the check to see how much T2D signal is present in the top PCs. Please also provide the relevant information needed to access the analysis code.**

Response: Thank you for the valuable feedback.

We have conducted a thorough investigation on how much T2D signal is presented in top PCs, which including the following steps and findings:

1. Among the top 20 PCs that we have adjusted for in our analysis, we examined their associations with T2D in UK Biobank. We found 5 PCs with significant associations with T2D (PC2, PC8, PC9, PC13, and PC14; p<0.0025).

2. We then performed genome-wide association analyses on each of these 5 PCs in UK Biobank and identified independent genome-wide significant variants associated with these PCs. The reviewer’s point was that potentially true genetic associations between these genetic variants and T2D will be attenuated after adjusting for PCs, even that it was acknowledged that this attenuation could be minimal.

3. Therefore, for genetic variants identified in step #2, we compared beta coefficients for T2D obtained in our UK Biobank analysis with estimates from the DIAGRAM GWAS consortia. The rationale to use the latest DIAGRAM GWAS as a reference was because this GWAS did not adjust for PCs in cohorts using BOLT-LMM and adjusted for the top 6 PCs in cohorts using logistic regressions (PMC6287706). As illustrated in the scatterplot below, beta coefficients for T2D were highly consistent between the two datasets, suggesting that adjusting for PCs did not attenuate association effects, even when they are associated with a PCs.

In terms of data and code sharing, we communicated with the Associate Editor on the proper procedures and have updated the Data Availability Statement accordingly.

We agreed on uploading scripts used to analyze UK Biobank data and the generation of the global polygenic score. Given the complexity of the computing, dataset management, and analytic systems in the Channing Division of Network Medicine, we agreed on describing how to access to the Channing system and locate the necessary files and code used to analyze the data from the Nurses’ Health Study I and II and the Health Professionals Follow up Study.

*The data underlying the generation of the global polygenic score for type 2 diabetes are available from the UK Biobank project site, subject to registration and application process. Further details can be found at* [*https://www.ukbiobank.ac.uk*](https://www.ukbiobank.ac.uk)*. This research was conducted under UK Biobank application no. 45052. Code to run the genome-wide association analysis for type 2 diabetes and generate the global polygenic score has been uploaded to (http://lianglab.rc.fas.harvard.edu/).*

*Information including the procedures to obtain and access the data and codes used in this study in the Nurses’ Health Study I and II, and the Health Professionals Follow-Up Study is described at http://www.nurseshealthstudy.org/researchers for the Nurses’ Health Study (contact: nhsaccess@channing.harvard.edu) or https://www.hsph.harvard.edu/hpfs/ for the Health Professionals Follow-up Study (contact: hpfs@hsph.harvard.edu).*

**2. Title: Please capitalize the first word of the subtitle, and please update this within the manuscript as well as the submission system: “Polygenic scores, diet quality, and type 2 diabetes risk: An observational study among 35,759 adults from three US cohorts”**

Response: Amended as suggested.

**3. Data availability statement: As mentioned by Reviewer 2, we request that you please make available the source code needed to replicate the study's findings in a repository (such as GitHub, SourceForge or Bitbucket) or a cloud computing service (such as Code Ocean). Protection of authors’ intellectual property will not be cause for exception. Please explain in the manuscript’s Data Availability Statement how readers can access the shared code and please also include an appropriate contact (web or email address) for inquiries (please note, this cannot be a study author).**

Response: Thank you for your guidance on this. We have updated the data availability statement accordingly.

**4. Abstract: Line 40-41: Please revise if this should be: “Health Professional’s Follow-up Study”**

Response: Thanks, we have revised this.

**5. Abstract: Line 53: We suggest revising to: “Limitations of this study include the self-report of diet information and possible bias resulting from inclusion of highly educated participants with available genetic data.”**

Response: Amended as suggested.

**6. Author summary: Line 63-64: Please revise to: “...the partial characterization of genetic risk and the predominant assessment of interactions on the multiplicative scale…” or similar.**

Response: Thanks for this suggestion.

**7. Author summary: Line 79-81: Please clarify if this should be: “Further, we showed that the risk of type 2 diabetes attributed to the combination of increased genetic risk and low diet quality was similar to the sum of the risks associated with each factor alone.”**

Response: Thanks for this suggestion.

**8. Methods: Line 116: Please change “RESEARCH DESIGN AND METHODS” to “Methods”**

Response: Amended as suggested.

**9. Methods: Line 252-253: Please describe how genetic risk categories (low, intermediate, and high) were established.**

Response: Thanks for this suggestion.

Page 14, line 252: “*We conducted analyses stratified by genetic risk category (low, intermediate, and high based on thirds of genetic risk distribution) to assess the association between diet quality and type 2 diabetes risk*.”

**10. References: Please check journal title abbreviations (for example, reference 5 should be N Engl J Med).**

Response: We have checked all journal tittle abbreviations.

**11. Table 1: How was race/ethnicity defined and by whom? Should the DASH scores be reported here, in addition to the AHEI?**

Response: Thanks for this suggestion. Race/ethnicity was self-reported in baseline questionnaires. Available options included non-Hispanic white (southern European/Mediterranean, Scandinavian, and other Caucasian ancestry), Black, Asian, American Indian, Hawaiian, or Hispanic. Hispanic and non-Hispanic whites were categorized as “White”, while Black, Asian, American Indian, and Hawaiian were categorized as “Others”. The rationale to combined Black, Asian, American Indian, and Hawaiian into a category call “Other” was due to the limited numbers of participants for these race/ethnicities. We have added the following information.

*Page 30, line 613: “Race was self-reported by the participants. Non-Hispanic white (southern European/Mediterranean, Scandinavian, and other Caucasian ancestry) and Hispanic were categorized into “White” while Black, Asian, American Indian, or Hawaiian were categorized into “Other”.”*

The DASH score was used in secondary analyses, and as a such it was included in Table 1.

**12. Table 3: The confidence interval for Beta-cell dysfunction is incomplete.**

Response: Thanks for this suggestion.

**13. Figure 1: Please note in the legend that IR is insulin resistance.**

Response: Amended as suggested.

**14. Figure 2: Please report p values as p<0.001 where relevant. Please report to two decimal places where p>/= 0.01 and please report to three decimal places where p<0.01.**

Response: Amended as suggested.

**15. Supporting Information File: Please provide a “clean” version of the document.**

Response: Thanks. We provide a clean version of the supplement in this iteration.

**16. S1 Fig: Please increase the font size of the axis labels on the graphs, if possible.**

Response: Amended as suggested.

**17. S2 Fig and S3 Fig: Please provide p values for the associations for each of the three cohorts.**

Response: Thank you for this suggestion. We have provided the p values for the association for each of the three cohorts.

**18. S4, S5, S6, S7, S8 Fig: Please report p values as p<0.001 where relevant. Please report to two decimal places where p>0.01 and please report to three decimal places where p<0.01.**

Response: We reported the p values as suggested.

**19. STROBE Checklist: Please provide a “clean” version of the checklist.**

Response: Thanks, we confirm we provided a clean version of the STROBE Checklist.

**Comments from Reviewers:**

**Reviewer #2: First of all, I apologize if I have been creating extra work for everybody involved. My original hope was that the authors would simply perform a quick check to see how much Type 2 Diabetes (T2D) signal is even present in the top principal components (PCs). Since PCs have to be computed for BOLT-LMM adjustment anyway, checking for correlation with T2D is a trivial amount of computation on top of that, and the very original comment could have been addressed with a single sentence to the effect of "To verify that adjusting for PCs did not remove a substantial amount of relevant signal, we examined stratification of patients into whether they have T2D by the top 20 PCs and found that <conclusion> (<relevant statistics here>)".**

**Instead, it appears that we are deep down the rabbit hole of discussing the merits of adjusting BOLT-LMM in general. To clarify, I agree with the authors that adjusting for the top PCs is a widespread practice, and that the resulting correction for possible confounders strongly outweighs any possible reduction in the signal of interest. I was simply suggesting to look at the amount of signal being lost (which was expected to be minimal), but this is a relatively minor sanity check that should not hold up the overall publication process. I also apologize if this didn't come across clearly in my earlier comments.**

**I can confirm that the financial disclosures statement correctly acknowledges relevant NIH grants. Unfortunately, the code is still being lumped under the data availability statement for some strange reason. I fully understand privacy concerns associated with releasing data, and the "available upon request" system is completely appropriate for it. However, computer code should not have the same constraints, and its release can help future researchers understand exactly what analyses were performed and with what parameters, thereby increasing overall reproducibility and robustness of the findings. It is a minimal amount of effort to upload existing analysis scripts to GitHub/GitLab or equivalent, add a simple README, and attach an open-source license (e.g., MIT or GPL). It is not entirely clear why the authors are so hesitant to publicly release their code, especially in an NIH-funded study, but this is not a hill that I am looking to die on with this manuscript.**

**Overall, I think this is a well-executed study, and the relatively minor points above should not prevent its progress towards a publication.**

Response: We sincerely thank the Reviewer for reviewing our paper and providing insightful comments. We have conducted a thorough investigation on how much T2D signal is presented in top PCs, which including the following steps and findings:

1. Among the top 20 PCs that we have adjusted for in our analysis, we examined their associations with T2D in UK Biobank. We found 5 PCs with significant associations with T2D (PC2, PC8, PC9, PC13, and PC14; p<0.0025).

2. We then performed genome-wide association analyses on each of these 5 PCs in UK Biobank and identified independent genome-wide significant variants associated with these PCs. The reviewer’s point was that potentially true genetic associations between these genetic variants and T2D will be attenuated after adjusting for PCs, even that it was acknowledged that this attenuation could be minimal.

3. Therefore, for genetic variants identified in step #2, we compared beta coefficients for T2D obtained in our UK Biobank analysis with estimates from the DIAGRAM GWAS consortia. The rationale to use the latest DIAGRAM GWAS as a reference was because this GWAS did not adjust for PCs in cohorts using BOLT-LMM and adjusted for the top 6 PCs in cohorts using logistic regressions (PMC6287706). As illustrated in the scatterplot below, beta coefficients for T2D were highly consistent between the two datasets, suggesting that adjusting for PCs did not attenuate association effects, even when they are associated with a PCs.

In terms of data and code sharing, we communicated with the Associate Editor on the proper procedures and have updated the Data Availability Statement accordingly.

We agreed on uploading scripts used to analyze UK Biobank data and the generation of the global polygenic score. Given the complexity of the computing, dataset management, and analytic systems in the Channing Division of Network Medicine, we agreed on describing how to access to the Channing system and locate the necessary files and code used to analyze the data from the Nurses’ Health Study I and II and the Health Professionals Follow up Study.

*The data underlying the generation of the global polygenic score for type 2 diabetes are available from the UK Biobank project site, subject to registration and application process. Further details can be found at* [*https://www.ukbiobank.ac.uk*](https://www.ukbiobank.ac.uk)*. This research was conducted under UK Biobank application no. 45052. Code to run the genome-wide association analysis for type 2 diabetes and generate the global polygenic score has been uploaded to (http://lianglab.rc.fas.harvard.edu/).*

*Information including the procedures to obtain and access the data and codes used in this study in the Nurses’ Health Study I and II, and the Health Professionals Follow-Up Study is described at http://www.nurseshealthstudy.org/researchers for the Nurses’ Health Study (contact: nhsaccess@channing.harvard.edu) or https://www.hsph.harvard.edu/hpfs/ for the Health Professionals Follow-up Study (contact: hpfs@hsph.harvard.edu).*
